# Effects of opium use on one-year major adverse cardiovascular events (MACE) in the patients with ST-segment elevation MI undergoing primary PCI: a propensity score matched - machine learning based study

**DOI:** 10.1186/s12906-023-03833-z

**Published:** 2023-01-19

**Authors:** Yaser Jenab, Behnam Hedayat, Amirali Karimi, Sarah Taaghi, Seyyed Mojtaba Ghorashi, Hamed Ekhtiari

**Affiliations:** 1 Professor of Cardiology, Fellowship of Interventional Cardiology, Tehran Heart Center, Tehran University of Medical Sciences, Tehran, Iran; 2 Assistant professor of Cardiology, Tehran Heart Center, Tehran University of Medical Sciences, Tehran, Iran; 3 School of medicine, Tehran University of Medical Sciences, Tehran, Iran; 4 Cardiologist, Tehran University of Medical Sciences, Tehran, Iran; 5 Cardiovascular Disease Research Institute, Tehran Heart Center, Tehran University of Medical Sciences, Tehran, Iran; 6 Department of Psychiatry, University of Minnesota, Minneapolis, MN USA; 7 Assistant professor of cardiology, Iran University of medical sciences, Tehran, Iran

**Keywords:** Machine learning, Major adverse cardiovascular events, Mortality,Myocardial infarction, Opium, Percutaneous coronary intervention

## Abstract

**Background:**

Considerable number of people still use opium worldwide and many believe in opium’s health benefits. However, several studies proved the detrimental effects of opium on the body, especially the cardiovascular system. Herein, we aimed to provide the first evidence regarding the effects of opium use on one-year major adverse cardiovascular events (MACE) in the patients with ST-elevation MI (STEMI) who underwent primary PCI.

**Methods:**

We performed a propensity score matching of 2:1 (controls: opium users) that yielded 518 opium users and 1036 controls. Then, we performed conventional statistical and machine learning analyses on these matched cohorts. Regarding the conventional analysis, we performed multivariate analysis for hazard ratio (HR) of different variables and MACE and plotted Kaplan Meier curves. In the machine learning section, we used two tree-based ensemble algorithms, Survival Random Forest and XGboost for survival analysis. Variable importance (VIMP), tree minimal depth, and variable hunting were used to identify the importance of opium among other variables.

**Results:**

Opium users experienced more one-year MACE than their counterparts, although it did not reach statistical significance (Opium: 72/518 (13.9%), Control: 112/1036 (10.8%), HR: 1.27 (95% CI: 0.94–1.71), adjusted *p*-value = 0.136). Survival random forest algorithm ranked opium use as 13th, 13th, and 12th among 26 variables, in variable importance, minimal depth, and variable hunting, respectively. XGboost revealed opium use as the 12th important variable. Partial dependence plot demonstrated that opium users had more one-year MACE compared to non-opium-users.

**Conclusions:**

Opium had no protective effects on one-year MACE after primary PCI on patients with STEMI. Machine learning and one-year MACE analysis revealed some evidence of its possible detrimental effects, although the evidence was not strong and significant. As we observed no strong evidence on protective or detrimental effects of opium, future STEMI guidelines may provide similar strategies for opium and non-opium users, pending the results of forthcoming studies. Governments should increase the public awareness regarding the evidence for non-beneficial or detrimental effects of opium on various diseases, including the outcomes of primary PCI, to dissuade many users from relying on false beliefs about opium’s benefits to continue its consumption.

**Supplementary Information:**

The online version contains supplementary material available at 10.1186/s12906-023-03833-z.

## Background

Opium was among the earliest plants used for its medicinal and recreational properties [1]. Derived from dried *Papaver somniferum L.* milky exudate, opium it is still ranked as the second common abused substance in the Middle East, just after tobacco [2]. This consumption trend is partly related to the proximity of this region to main production centers of opium, causing easier accessibility to this drug. Although the more conventional opium use have lost its popularity in many world regions, the family of opioids account for the highest share of disease burden related to illicit drug use worldwide [3].

Many people who use opium believe in its protective effects against diseases, including cardiovascular morbidity, and such beliefs may account for the tendency towards opium or reluctance to give up its use [4, 5]. Nevertheless, the studies oppose such claim. For instance, a meta-analysis of 41 studies found a 2.75 (95% confidence interval (CI): 2.04–3.75) increased risk of coronary artery disease (CAD) in patients who use opium [6]. Several other studies also announced opium as a risk factor for increased cardiovascular and all-cause mortality [4–12]. Opium can exert its detrimental effects via numerous mechanisms, such as increasing inflammation, coagulation, and oxidative stress, decreasing physical activity, and adverse hormonal and metabolic changes, etc., that are further expanded in the discussion [1].

Earlier researchers studied the effects of opium on patients undergoing coronary artery bypass graft (CABG) surgery and observed the adverse outcomes of patients who use opium in this setting [13, 14]. In one study the patients who used opium had higher 5-year major adverse cardiovascular events (MACE) and mortality [14], while in the other they had higher readmission rates [13]. However, no evidence exists regarding the effects of opium on the outcomes of patients undergoing primary percutaneous coronary intervention (PCI) after ST-segment elevation myocardial infarction (STEMI). Only one study exists in the elective PCI settings, but found no associations between opium use and one-year MACE. Therefore, we aimed to study one-year outcomes of these patients using conventional statistical analysis and machine learning strategies.

## Methods

### Study population

We conducted a retrospective cohort study to assess the effect of opium use and cardiovascular outcome in the first year after primary PCI for STEMI patients. A total of 3466 patients who underwent primary PCI were initially included in this study, including 586 opium users and 2922 non-opium users as controls.

Patients’ data was extracted from Tehran Heart Center Primary PCI database. The percentage of missing values were evaluated after dataset’s variables modifications. Variables with missing values of more than 10% were excluded. For conventional analysis, remaining missing values were imputed by replacing the value with mode and median for the categorical and numerical values, respectively. We chose median because the base analysis demonstrated the distribution of all the numeric variables were not normal. Then, we performed 2:1 propensity score matching (PSM) yielding 518 opium users and 1036 controls and all the analyzes in this study, including both statistical and machine learning methods, were performed on these matched groups. All the analyzes were carried out using R statistical packages v4.0.4 (http://www.r-project.org/).

### Baseline characteristics and propensity score matching (PSM)

To compare baseline characteristics between the opium users and control groups, student t-test and Mann-Whitney U-test were used for numeric variables with normal and non-normal distributions, respectively, and Chi-square test was used for categorical variables. Numeric variables with normal distribution were reported with mean and 95% confidence interval (CI) and numeric variable with non-normal distribution were reported with median and interquartile range (IQR). Categorical variables were reported with count and percentage. Two-sided alpha value of 0.05 was considered as significant level. Supplementary Table 1 shows the between-group differences of baseline characteristics before and after matching. Only four variables of body mass index (BMI) (opium: Median: 27 vs. control: 27.11), triglyceride (116 vs. 125), creatinine (1.0 vs. 0.9), and hemoglobin (15 vs 15.7) remained statistically significant between the groups, but their differences were clinically insignificant.

PSM was conducted to minimize differences in baseline propensity of observations to be assigned to the independent variable of interest, opium use. PSM was conducted by the logistic regression method with 2:1 matching (2 control: 1 opium). Greedy nearest-neighbor method without replacement was performed to choose nearest distance of each observation propensity score in opium user and control groups.

Variables included in the matching process were selected based on the baseline characteristics comparison results, those with statistically and clinically significant difference were included in a logistic regression model. There was a significant difference between opium users and control group in baseline prevalence of hypertension, diabetes mellitus (DM), dyslipidemia, smoking history, gender, and baseline mean of fasting blood sugar, age, and low-density lipoprotein (LDL) levels (Table 1).

**Table 1:** Variables included in logistic regression to calculate propensity scores of observations in opium users and control groups, before and after matching

		Before Matching			After Matching		
**Variable**	**Category**	**Opium use**	**Control**	***p***-value	**Opium use**	**Control**	***p***-value
Hypertension				<0.001*			0.9
	No	*n* = 341 (65.8%)	*n* = 1505 (52.8%)		*n* = 341 (65.8%)	*n* = 677 (65.3%)	
	Yes	*n* = 177 (34.2%)	*n* = 1343 (47.2%)		*n* = 177 (34.2%)	*n* = 359 (34.7%)	
DM				<0.001*			1
	No	*n* = 354 (68.3%)	*n* = 1623 (57%)		*n* = 354 (68.3%)	*n* = 708 (68.3%)	
	Yes	*n* = 164 (31.7%)	*n* = 1225 (43%)		*n* = 164 (31.7%)	*n* = 328 (31.7%)	
Hyperlipidemia				<0.001*			0.73
	No	*n* = 292 (56.4%)	*n* = 1306 (45.9%)		*n* = 292 (56.4%)	*n* = 573 (55.3%)	
	Yes	*n* = 226 (43.6%)	*n* = 1542 (54.1%)		*n* = 226 (43.6%)	*n* = 463 (44.7%)	
FBS		106 (IQR: 93–136)	115 (IQR: 98–157)	< 0.001*	106 (IQR: 93–136)	111 (IQR: 96–137.5)	0.08
LDL		94 (IQR: 74–120.75)	101 (IQR: 79–122)	0.003*	94 (IQR: 74–120.75)	100 (IQR: 76–119)	0.38
Gender				< 0.001*			0.32
	Female	*n* = 19 (3.7%)	*n* = 731 (25.7%)		*n* = 19 (3.7%)	*n* = 27 (2.6%)	
	Male	*n* = 499 (96.3%)	*n* = 2117 (74.3%)		*n* = 499 (96.3%)	*n* = 1009 (97.4%)	
Age		58 (IQR: 52–65)	61 (IQR: 53–69)	<0.001*	58 (IQR: 52–65)	58 (IQR: 51–65.25)	0.96
Chronic smoking				<0.001*			0.66
	No	*n* = 91 (17.6%)	*n* = 1758 (61.7%)		*n* = 91 (17.6%)	*n* = 193 (18.6%)	
	Yes	*n* = 427 (82.4%)	*n* = 1090 (38.3%)		*n* = 427 (82.4%)	*n* = 843 (81.4%)	

After applying PSM, absolute standardized mean difference (SMD) plot of the variables included in PSM demonstrated perfect matching of the selected variables as all the SMDs reduced to less than 10% (Supplementary Fig. 1).

### Conventional statistical analysis

Univariate cox regression analysis was performed for each of the independent variables, as follows:

$$\lambda \left(\left.t\right|x\right)={\lambda}_0(t)s(x)$$

where *s(x)* is relative risk function, *λ*_0_*(t)* is baseline hazard,* λ(t|x)* is hazard function *λ* at time of *t* for an observation with covariate vector *x* is calculated.

Variables with significant *p*-value of less than 0.1 in each model and their model Wald test *p*-value of less than 0.1 were selected for multivariate cox regression analysis. Opium was included in the multivariate analysis regardless of its significant level in the univariate analysis. Assessment of proportionality of hazard function was assessed by Shoenfeld’s residuals. None of the predictors violated proportionality of hazard functions.

Hazard ratio (HR) for opium use was calculated in the multivariate analysis and then, Kaplan-Meier (KM) curves were plotted for one-year mace MACE and its components (all-cause mortality, myocardial infarction (MI), target vessel revascularization (TVR), target lesion revascularization (TLR), and CABG).

### Machine learning analysis

We conducted machine learning analysis as a sensitivity analysis to assess robustness of the results. Two infamous machine learning algorithms, Survival Random Forest and Extended Gradient Boosting for survival study (XGboost), with built-in variable importance and feature selection capability were selected. We used mlr3proba 0.4.0 version and its dependent packages (mlr3extralearner, mlr3pipelines, mlr3filter, etc.) for implementing machine learning algorithms in the R software. The advantage of these packages lies is their capability of implementing machine learning algorithms for survival studies which have different structure from classification studies because of counting time in addition to events.

#### Data splitting

The main dataset with missing values was randomly split into train and test parts with 80 and 20% of the total data, respectively. All hyperparameter optimization, training and benchmarking processes were conducted on the train set. Final assessment of the model’s accuracy was conducted on the test set.

#### Data pre-processing

Categorical predictors were transformed to numerical using numerical encoding. To prevent data leakage, missing values were replaced in training and test datasets separately by median and mode for numerical and categorical variables, respectively. Median (IQR) was chosen due to non-normal distribution of both groups. To avoid significant multi-collinearity between numeric variables, correlation matrix of independent numerical variables was assessed and a correlogram was plotted. The degree of collinearity between two variables by Pearson correlation coefficient was considered weak if 0 ≤ |*r*| < 0.3, moderate if 0.3 ≤ |*r*| < 0.7 and strong if |*r*| ≥ 0.7 [15]. Total cholesterol and LDL had significant co-linearity, so total cholesterol was dropped from features (Supplementary Fig. 2).

#### Base learner

Decision Tree is one of the well-known algorithms of machine learning, constructed from nodes and leaves. It divides subjects by input features according to their outcomes until best separation of observations with homogeneous survival outcomes achieves. Decision tree is the default base-learner (weak-learner) of survival random forest algorithm. Splitting rule for studies which all observation may not have complete follow-up, as in survival studies, would be different from classification problems.

Two main methods of splitting nodes to daughter’s nodes in survival decision trees are node purity and node distance methods. Default splitting rule of decision trees of survival random forest of mlr3proba package is “log-rank” hypothesis tests which is a “node distance based” splitting-rule. Briefly describing, the null-hypothesis of log-rank test assumes that survival distribution and hazard functions of two separate groups of observations are identical. Here the algorithm performs log-rank test in each split, comparing hazard function of two leaves.

Considering *h*^*A*^ as leaf “A” hazard function and *h*^*B*^ as leaf B hazard function, then log-rank null and alternative hypothesis would be:

$${H}_0:{h}^A={h}^B$$

$${H}_1:{h}^A\ne {h}^B$$

respectively, and assuming:

**Table:** 

${d}_{\tau}^A$	No. of observed deaths in leaf A at time *τ*
${e}_{\tau}^A$	No. of expected deaths in leaf A at time *τ*
${\upsilon}_{\tau}^A$	Variance of the No. of deaths in leaf A at time *τ*
*υ*_*D*_	list of unique event times in both leaves

Then, log-rank statistics would be [16]:

$$LogRank\left({leaf}^A\right)=\frac{\sum_{\tau \in {\upsilon}_D}\left({d}_{\tau}^A-{e}_{\tau}^A\right)}{\sqrt{\sum_{\tau \in {\upsilon}_D}{\upsilon}_{\tau}^A}}$$

The result of log-rank test indicates degree of dissimilarity between two leaves in each split. The higher its score, the more different is hazard functions of leaves, hence more discriminative is the feature in the splitting process.

Default splitting rule of decision trees of XGboost algorithm of mlr3proba package is full likelihood deviance measures of cox model, which we used in in our study. It is based mainly on estimating cumulative hazard function of each node by Cox model, and trying to maximize full proportional hazard likelihood. As it is discussed by LeBlanc and Crowely [16], it tries to maximized reduction in one-step deviance.

As a brief description, considering following definition:

$\overset{\sim }{T}$as a set of terminal nodes,

*S*_*h*_ as a set of observation labels in terminal node *h*,

*λ*_*0*_ as hazard function,

*Λ*_*0*_(*t*) as baseline cumulative hazard function,

*t*_*i*_ observation time of individual *i*,

*δ*_*i *_failure indicator for individual *i*, which would be zero or one,

Then full likelihood score of node *h* given tree *T* would be:

1 $$L={\prod}_{h\in \overset{\sim }{T}}{\prod}_{i\in {S}_h}{\left({\lambda}_0\left({t}_i\right){\theta}_h\right)}^{\left({\delta}_i\right)}{e}^{-{\varLambda}_0\left({t}_i\right){\theta}_h}$$

Then deviance of node *h* would be the difference between fitted model and saturated model maximum log-likelihood values:

$$R(h)=2\left\{{loglikelihood}_h(saturated)-{loglikelihood}_h\left({\overset{\sim }{\theta}}_h\right)\right\}$$

where $loglikelihood\left(\overset{\sim }{\theta_h}\right)$ is the maximized log-likelihood and the baseline cumulative hazard function *Λ*_0_(*t*) is known.

The deviance residual of node *h* in terms of proportional hazard function would be:

$$R(h)=\frac{1}{N}{\sum}_{i\in {S}_h}\left[{\delta}_i\mathit{\log}\left(\frac{\delta_i}{\hat{\Lambda_0^1}\left({t}_i\right)\hat{\theta_h}}\right)-\left({\delta}_i-\hat{\Lambda_0^1}\left({t}_i\right)\hat{\theta_h}\right)\right]$$

Therefore, the improvement of deviance of node *h* into left daughter nodes *l*_*node*_(*h*) and right daughter nodes *r*_*node*_(*h*) is

$$R\left( split,h\right)=R(h)-\left[R\left({l}_{node}(h)\right)+R\left({r}_{node}(h)\right)\right]$$

The algorithms perform binary splitting with all possible split of covariates to achieve maximum reduction in deviance measures in each split.

The default evaluation metrics of consecutive trees for survival XGboost algorithm in our study, was cox-nlog-likelihood.

Supplementary Fig. 3 illustrates two of the decision trees plotted in our study as an example.

#### Ensemble methods

The main advantage of decision tree is its low bias rates compared to other base-learners, but it has high variance. To reduce its variance, ensemble methods have been developed to aggregate the results of many trees and improve the prediction. We used survival random forest and XGboost (extended gradient boosting) for survival analysis as ensemble methods. Random forest utilizes a bagging (bootstrap aggregating) method and XGboost follows a gradient boosting algorithm.

#### Hyperparameter optimization

Important machine learning algorithms’ hyperparameters must be tuned before implementing the final model on the new test datasets. We utilized “random-search” tuning strategy with terminating rule defined as 50 iterations to optimize hyperparameters of each the algorithms.

Important hyperparameters for random forest algorithms were assessed for optimization and those contributing to improved model’s accuracy, were provided to the algorithm, including number of variables to choose randomly in each splits (“mtry”) and minimum number of objects in each terminal node(“nodesize”) (Table 2). In a spot check assessment of data, the out-of-bag error rate of survival random forest stabilized after about 250–300 trees (Supplementary Fig. 4), so we defined 1000 trees as number of trees to be generated for random forest.

**Table 2:** Results of hyperparameter Optimization. The hyperparameters are sorted from lowest to highest based on the resulting model’s accuracy in each step

Survival Random Forest	Value	Accuracy	Survival XGboost	Value	Accuracy
No. of trees	1000	62.5%	No. of consecutive trees	195	58.8%
No. of variables in each split	5	63.7%	Maximum depth of tree	15	60.6%
Min No. of objects in each terminal node	27	65%	Minimum child weight	7	61.8%
			Gamma	0.35	63.5%
			Eta	0.1	64%

For XGboost algorithm, eleven hyperparameters were assessed for optimization (including nrounds, max_depth, min_child_weight, etc.) and those contributing to improved model’s accuracy were provided to the algorithm.

**Fig. 1:**
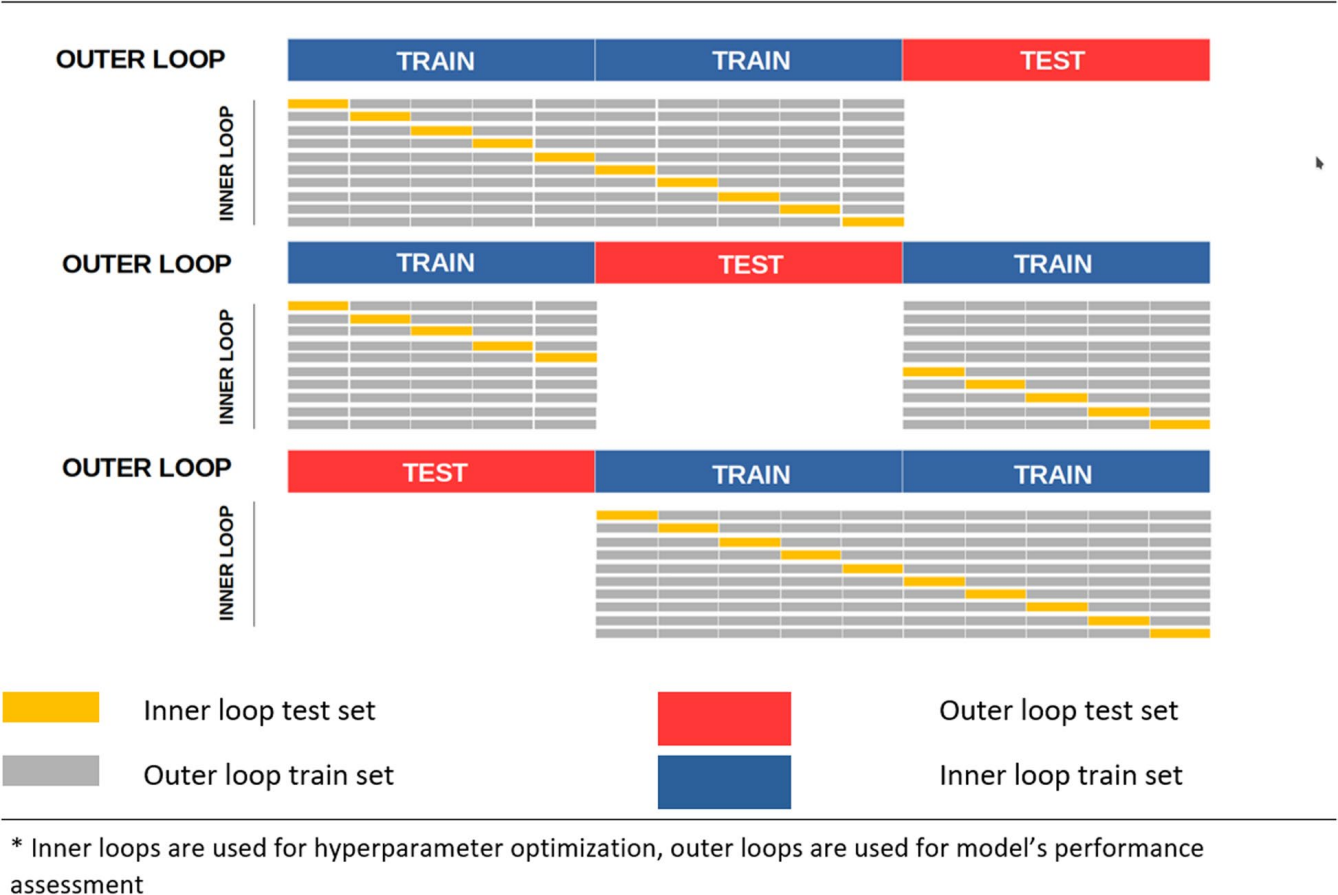
Nested cross validation for hyperparameter optimization of XGboost model with 3 outer resampling loop and 10 inner resampling loop

**Fig. 2:**
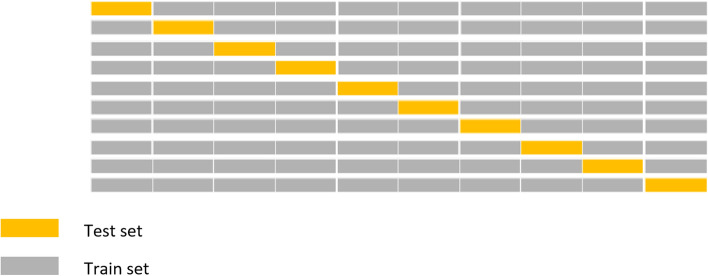
Ten-folds cross validation for hyperparameter optimization of survival random forest model

To reduce the probability of data leakage and over-fitting during optimization, “nested cross-validation” with 10 inner folds and 3 outer folds was conducted to assess the improved accuracy of XGboost model by each hyperparameters (Fig. 1). For the survival random forest, simple 10 folds cross-validation was used (Fig. 2).

#### Benchmarking

To compare machine learning (ML) algorithms with reliability, it is necessary to ensure that train and test datasets are the same for all of the algorithms. With Benchmarking, one can apply resampling methods on main dataset, assuring all algorithms are being implemented on exactly the same train/test set in each resampling run. Then, we extract the overall average results of resampling in each set of populations.

We included non-tree-based cox proportional hazard model (Although, this time with full set of features) in benchmark resampling to enable the comparison of our two ML methods (i.e Survival Random Forest, XGboost) with traditional cox proportional hazards method used in our study. We conducted benchmark resampling using 3-folds cross-validation.

Two measurement indices for survival studies were selected, Harrell’s C-index and Uno’s C-index to compare the algorithms. These are somehow equivalent to area under the curve (AUC) used for classification algorithms.

Harrell’s C-index, is an algorithm which is used to assess time to event studies performance. The main concept behind Harrell’s C-index is that a pair of subjects with different time of event experience, so called “comparable” subjects according to time of event, would have different calculated risk of experiencing event. The less time an event occurs, the higher the risk one subject would have. Therefore, a “comparable” subject’s pair risk estimation is expected to be “concordant” to their time of events.

Harrell’s C-index calculates ratio of “concordant” to “comparable” pairs, meaning how much the model has accurately measure the risks that are concordant to time of events [17].

$$\hat{C}=\frac{\sum_{i=1}^N{\varDelta}_i{\sum}_{j=i+1}^NI\left({T}_i^{obs}<{T}_j^{obs}\right)I\left({M}_i>{M}_j\right)}{\sum_{i=1}^N{\varDelta}_i{\sum}_{j=i+1}^NI\left({T}_i^{obs}<{T}_j^{obs}\right)}$$

In the above equation, ${T}_i^{obs},{T}_j^{obs}$ are time to event for observation i and observation j respectively, and ${M}_i^{obs},{M}_j^{obs}$ are calculated risks for observation i and observation j. The result of *I*(…) would be zero or one according to comparison results.

#### Features ranking

Results of training of the train set were used to conduct variable importance. For random forest, out-of-bag samples during creating trees were used for variable importance method.

For survival random forest, we utilized permutation variable importance (VIMP) as described by Breiman et al. [18], tree minimal depth methodology, and variable hunting to rank variables based on their level of importance.

Permutation importance assesses model accuracy (error rate) before and after permuting (random shuffling) of each variable; the more deterioration occurs in the model accuracy, the more important permuted variable is.

As described by Breiman et al. [18], considering following definitions:

*t*: one of the trees where *t* ∈ {1, …, *ntree*.}

${\overline{B}}^{(t)}$: out of bag (oob) sample for *t*

*X*_*j*_: variable *j* in tree *t*

${\hat{y}}_i^{(t)}={f}^{(t)}\left({x}_i\right)$: predicted value of observation *i* before permutation of its value of *X*_*j*_

${\hat{y}}_{i,{\pi}_j}^{(t)}={f}^{(t)}\left({x}_{i,{\pi}_j}\right)$: predicted value of observation *i* after permutation of its value of *X*_*j*_

Then variable importance of *X*_*j*_ in tree *t* is calculated as follows:

2 $${VI}^{(t)}\left({X}_j\right)=\frac{\sum_{i\in {\overline{B}}^{(t)}}I\left({y}_i={\hat{y}}_i^{(t)}\right)}{\left|{\overline{B}}^{(t)}\right|}-\frac{\sum_{i\in {\overline{B}}^{(t)}}I\left({y}_i={\hat{y}}_{i,{\pi}_j}^{(t)}\right)}{\left|{\overline{B}}^{(t)}\right|}$$

Then for each variable, mean variable importance score is calculated as follows:

$$VI\left({X}_j\right)=\frac{\sum_{t=1}^{ntree}{VI}^{(t)}\left({X}_j\right)}{ntree}$$

which is the mean variable importance score of v among all trees.

For minimal depth method, a preliminary random forest is generated first, then VIMP of each variable is calculated and is used to weigh each variable. Then routine random forest run is conducted but this time instead of randomly selecting variables in each node split, they are selected with a chance that is proportional to their assigned weights. It searches subtrees which their root nodes are split by variable *v*, so called maximal subtrees of variable *v*. A closest maximal subtree root of variable *v* to the main tree root is called minimal depth of variable *v*. The smaller minimal depth, the more important the variable *v* in predicting the outcome.

We used 50 iterations of survival random forest for minimal depth method (using package ‘randomForestSRC’ Hemant Ishwaran, version 3.1.1).

Variable hunting (VH) method usually is implemented for high-dimensional dataset (number of variables remarkably more than subjects, e.g. 10 times), our dataset was not high-dimensional, but we used this method to investigate the concordance between all methods of variable importance.

VH method in randomForestSRC package (one of mlr3proba dependency) follows this sequence: A preliminary forest is created to calculate VIMP of each variable, then another forest is created by selecting variables with chance proportional to their VIMP (weight). But this time instead of “depth”, relative frequency of selecting a variable is used to determine its importance, the more the relative frequency is, the more the variable is important. We defined 50 numbers of survival random forest iterations, and one preliminary tree was created before each iteration to calculate VIMP scores [using package ‘randomForestSRC’ Hemant Ishwaran, version 3.1.1]. Again, All VIMP scores were calculated by Breiman-Cutler permutation.

For XGboost algorithm, we used built-in “feature importance” function of XGboost package, which calculate the relative number of a feature that selected for splitting nodes across all trees, and percentage of total gain increase in all splits of a feature.

### Partial dependence plot (PDP)

We used partial dependence to assess the average marginal effect of selected top features on the target variable, MACE. For numerical features, it helps to find the pattern of relation between the features and outcome, as they are linear or non-linear. For categorical variables it helps to compare effects of each category on the target variable.

For numerical variables, considering *x*_*S*_ as the feature(s) in the set *S* that we want to plot its/their relation(s) with the outcome variable, *x*_*C*_ as vector of other features used in our ML model $\hat{f}$, partial function marginalizes ML output over various distributions of vector *x*_*C*_ variables, so the function would depends mainly on *x*_*S*_, our variable of interest [19]:

$$\hat{f}_{x_S}\left({x}_S\right)=\int \hat{f}\left({x}_S,{x}_C\right) dP\left({x}_C\right)$$

And estimation of partial function $\hat{f}_{x_S}$ by averaging marginal effects as [19]:

$$\hat{f}_{x_S}\left({x}_S\right)=\frac{1}{n}{\sum}_{i=1}^n\hat{f}\left({x}_S,{x}_C^{(i)}\right)$$

To compute the marginal effect of a categorical variable, we set the category of all observations to the category that we are interested. For example, considering hypertension as a variable with two categories of 0 and 1, we calculate PDP estimate of having and not having hypertension (i.e. 1 or 0). Then we replace hypertension status of all the observations to 1 at once, and perform prediction, and then to 0 and perform the prediction again  [19].

### Web application and source codes

To demonstrate applicability of our study, we developed a web application which can be used to predict first year MACE of primary PCI patients by uploading proper data file by users or real-time completing a form of features (webapp link: https://behnam-hedayat.shinyapps.io/primace or https://primace.aikadeh.com). The source code of statistical and machine learning analysis and the web application  are available in Supplementary Table 3.

## Results

### Conventional statistical analysis

**Fig. 3:**
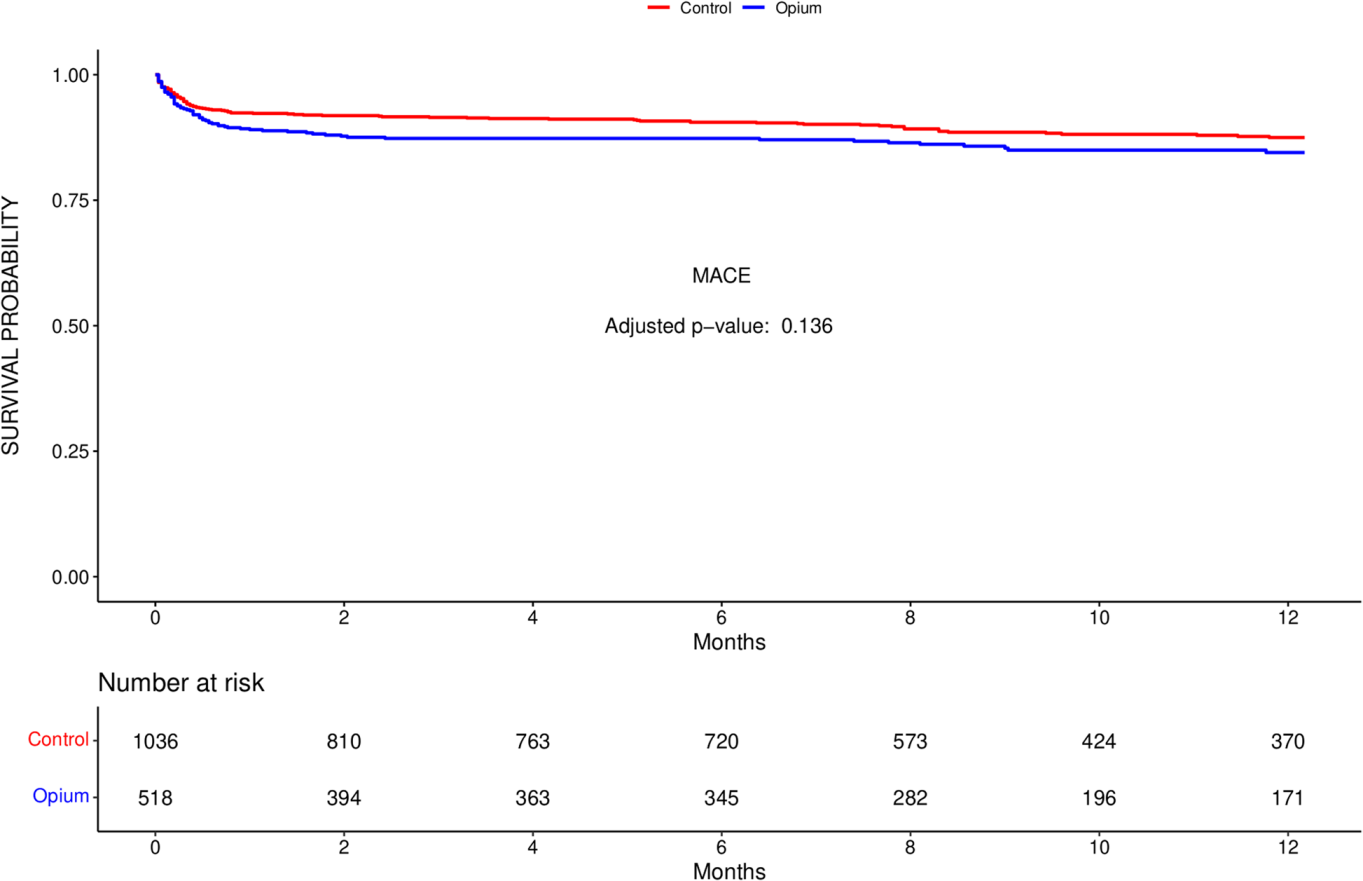
Kaplan–Meier (KM) curves of one-year MACE of the patients who underwent primary PCI after ST-segment elevation MI separated by opium users and controls

Opium users experienced about 27% more MACE during one-year after primary PCI compared to their counterparts, although that was not proved to be significant in multivariate cox regression model (Opium: 72/518 (13.9%), Control: 112/1036 (10.8%), HR: 1.27 (95% CI: 0.94–1.71), adjusted *p*-value = 0.136) (Table 3). KM curves were plotted for one-year MACE (Fig. 3) and its components (Supplementary Fig. 5), all without significant differences between the groups. One-year need for CABG after primary PCI was the most notably different component of MACE between the groups, although it was not significantly changed, but it suggests a trend toward more one-year need for CABG in patients who used Opium compared to non-users (HR: 1.56 (95% CI: 0.98–2.5), adjusted *p*-value = 0.063) (Table 4).

**Table 3:** Results of Cox regression MACE analysis for different variables, including opium use

		Univariate			Multivariate	
**Variable**	**Category**	**HR (CI 95%)**	***p***-value	Wald ***p***-value	**HR (CI 95%)**	***p***-value
Clopidogrel use				0.041 *		
	Yes	1.362 (1.015–1.828)	0.039 *		1.51 (0.95–2.39)	0.081
Pain to Door time		1 (0.999–1)	0.053 *	0.028 *	1 (1–1)	0.028 *
Calcium channel blocker use				0.368		
	Yes	1.381 (0.707–2.698)	0.345			
Nitrate use				0.138		
	Yes	1.254 (0.932–1.687)	0.134			
HDL		0.993 (0.977–1.009)	0.393	0.389		
FBS		1.004 (1.002–1.005)	<0.001*	<0.001*	1 (1–1)	0.025 *
LDL		0.996 (0.992–1.001)	0.132	0.128		
Triglyceride		0.999 (0.997–1.001)	0.243	0.215		
BMI		0.981 (0.947–1.016)	0.273	0.27		
Creatinine		1.203 (1.074–1.349)	0.001 *	0.014 *	1.24 (1.08–1.42)	0.002 *
Hemoglobin		0.837 (0.778–0.901)	<0.001*	<0.001*	0.89 (0.82–0.97)	0.006 *
Statin use				0.21		
	Yes	1.204 (0.901–1.608)	0.209			
COPD				0.677		
	Yes	0.754 (0.187–3.038)	0.691			
Aspirin use				0.09 *		
	Yes	1.288 (0.96–1.727)	0.092 *		0.76 (0.49–1.18)	0.219
Beta blocker use				0.125		
	Yes	1.259 (0.94–1.685)	0.122			
ACEI/ARB use				0.055 *		
	Yes	1.328 (0.994–1.773)	0.055 *		0.86 (0.56–1.33)	0.499
Hypertension				0.009 *		
	Yes	1.485 (1.109–1.987)	0.008 *		1.3 (0.94–1.8)	0.118
DM				0.004 *		
	Yes	1.553 (1.158–2.083)	0.003 *		1.12 (0.76–1.64)	0.567
Hyperlipidemia				0.917		
	Yes	0.985 (0.736–1.317)	0.917			
Family history of MI				0.198		
	Yes	0.761 (0.496–1.169)	0.213			
IHD				0.002 *		
	Yes	1.623 (1.207–2.182)	0.001 *		1.47 (1.06–2.04)	0.023 *
Final TIMI2 score				<0.001*		
	1	0.37 (0.25–0.548)	<0.001*		0.64 (0.4–1.02)	0.059
Gender				0.084 *		
	Male	0.523 (0.268–1.022)	0.058 *		0.78 (0.38–1.6)	0.499
Age		1.029 (1.016–1.042)	<0.001*	<0.001*	1.02 (1–1.03)	0.014 *
Chronic smoking				0.059 *		
	Yes	0.713 (0.506–1.003)	0.052 *		0.85 (0.59–1.22)	0.385
Initial TIMI2 score				0.043 *		
	1	0.79 (0.455–1.372)	0.403		0.79 (0.45–1.39)	0.416
	2	0.644 (0.428–0.969)	0.035 *		0.62 (0.41–0.95)	0.027 *
	3	0.556 (0.308–1.004)	0.052 *		0.56 (0.31–1.02)	0.058
PCI Result				<0.001*		
	Unacceptable	3.528 (2.344–5.308)	<0.001*		2.66 (1.63–4.35)	<0.001*
Culprit vessel				0.454		
	LADnp	0.774 (0.506–1.182)	0.236			
	non. LAD	0.951 (0.682–1.326)	0.767			
IABP				0.034 *		
	Yes	7.339 (1.82–29.589)	0.005 *		2.75 (0.64–11.83)	0.174
Cardiogenic shock				0.073 *		
	Yes	12.528 (1.75–89.672)	0.012 *			
Door to Device time		0.999 (0.997–1.001)	0.311	0.263		
GPIIbIIIa inhibitors use				0.918		
	Yes	1.017 (0.732–1.414)	0.918			
Opium use				0.074 *		
	Yes	1.314 (0.977–1.767)	0.071 *		1.27 (0.94–1.71)	0.125

Abbreviations: *HR *Hazard ratio, *CI *Confidence interval, *HDL *High-density lipoprotein, *FBS *Fasting blood sugar, *LDL *Low-density lipoprotein, *BMI *Body mass index, *COPD *Chronic obstructive pulmonary disease, *ACEI *Angiotensin-converting enzyme inhibitor, *ARB *Angiotensin receptor blocker, *DM *Diabetes mellitus, *MI *Myocardial infarction, *IHD *Ischemic heart disease, *TIMI *Thrombolysis in Myocardial Infarction, *PCI *Percutaneous coronary intervention, *LAD *Left anterior descending coronary artery, *LADnp *Non-proximal LAD, *IABP *Intra-aortic balloon pump

* Wald *p*-value of less than 0.1 was the cut-off point for univariate analysis. Significant level for the multivariate analysis was *p* < 0.05

**Table 4:** Effect of opium consumption on different one-year outcomes after primary PCI

Outcome	Opium	Control	HR (CI %95)	*p*-value
**MACE**	72/518 (13.9%)	112/1036 (10.81%)	1.26 (0.93–1.7)	0.136
**All-cause Mortality**	23/518 (4.44%)	41/1036 (3.96%)	1.07 (0.64–1.81)	0.795
**MI**	12/518 (2.32%)	23/1036 (2.22%)	1.04 (0.51–2.08)	0.923
**TVR**	1/518 (0.19%)	1/1036 (0.1%)	0.85 (0.04–16.72)	0.912
**TLR**	4/518 (0.77%)	7/1036 (0.68%)	1 (0.29–3.48)	0.998
**CABG**	32/518 (6.18%)	40/1036 (3.86%)	1.56 (0.97–2.49)	0.065

Abbreviations: *HR *Hazard ratio, *CI *Confidence interval, *MACE *Major adverse cardiovascular events, *MI *Myocardial infarction, *TVR *Target vessel revascularization, *TLR *Target lesion revascularization, *CABG *Coronary artery bypass graft

*Significant level was p < 0.05

### Machine learning analysis

#### Random forest results

**Fig. 4:**
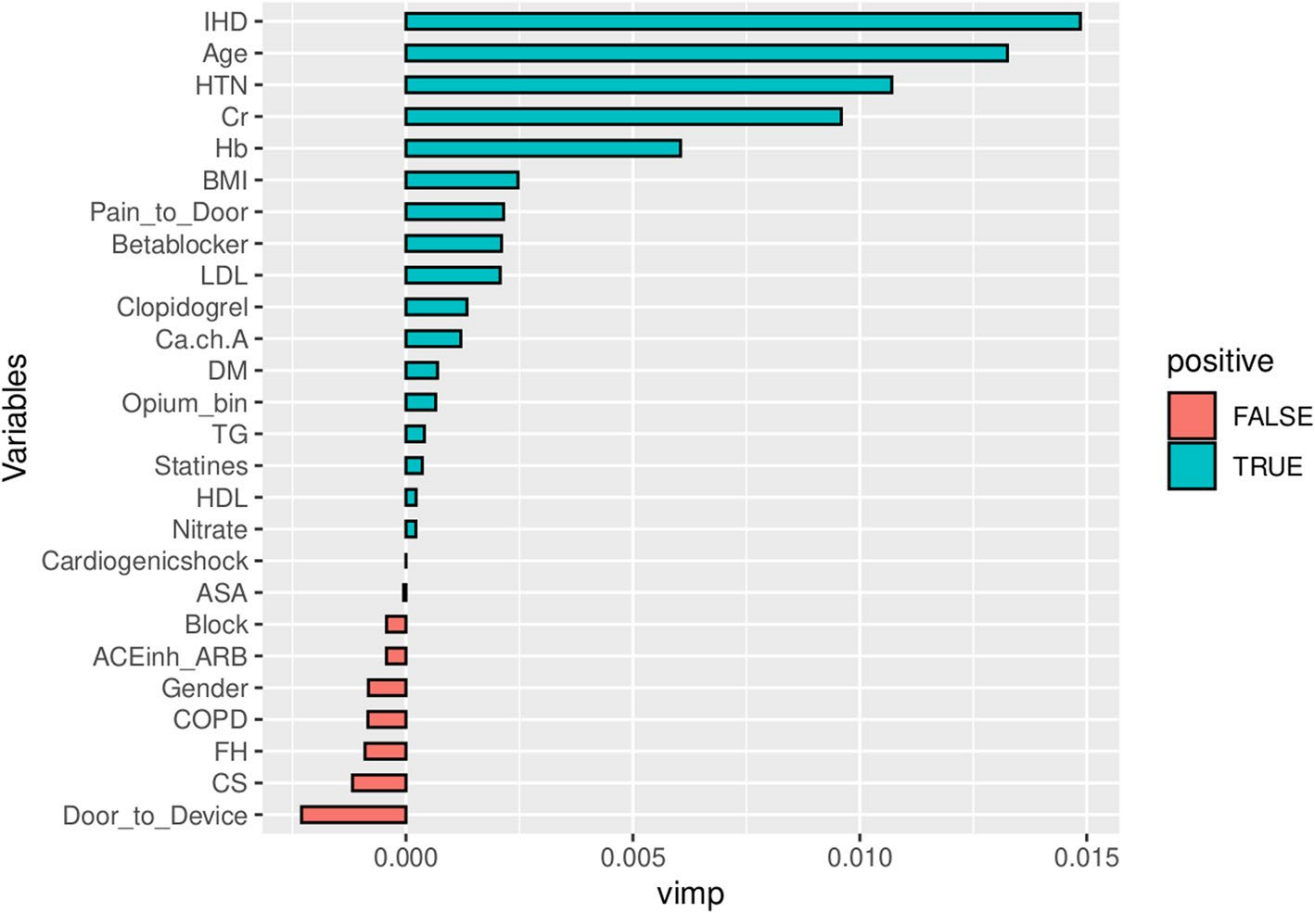
Variable importance analysis by minimal depth method

**Fig. 5:**
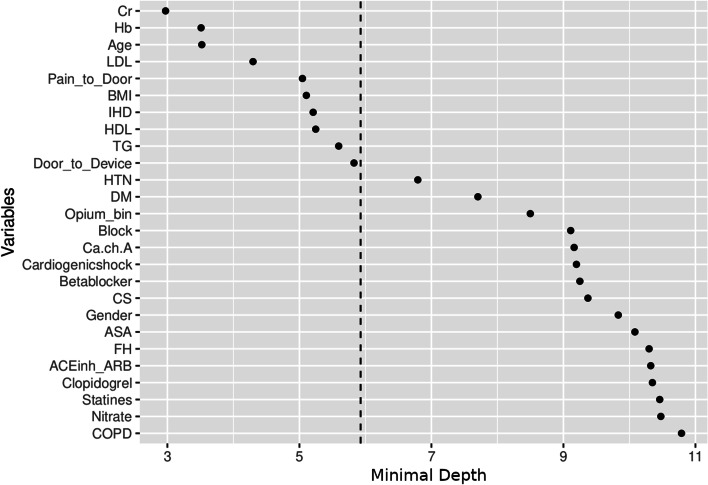
Feature selection by Random Forest algorithm

**Fig. 6:**
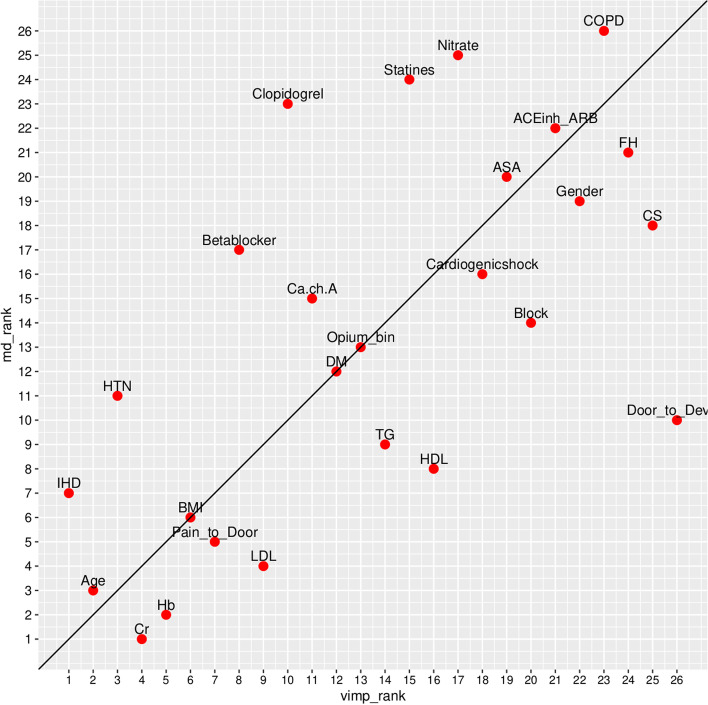
Variable importance vs. feature selection by minimal depth rankings of the included variables

**Fig. 7:**
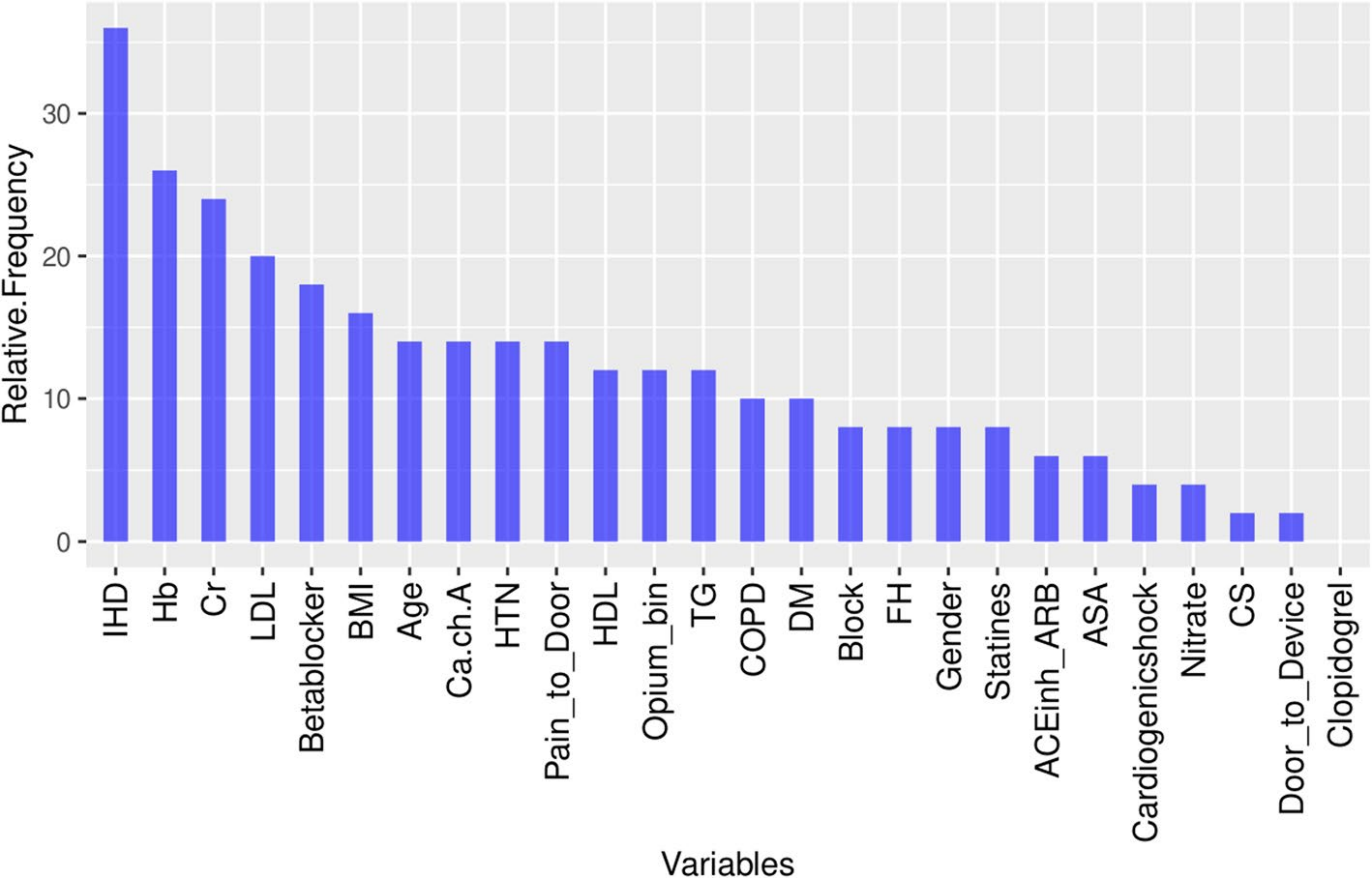
Variable hunting analysis by Random Forest algorithm

**Fig. 8:**
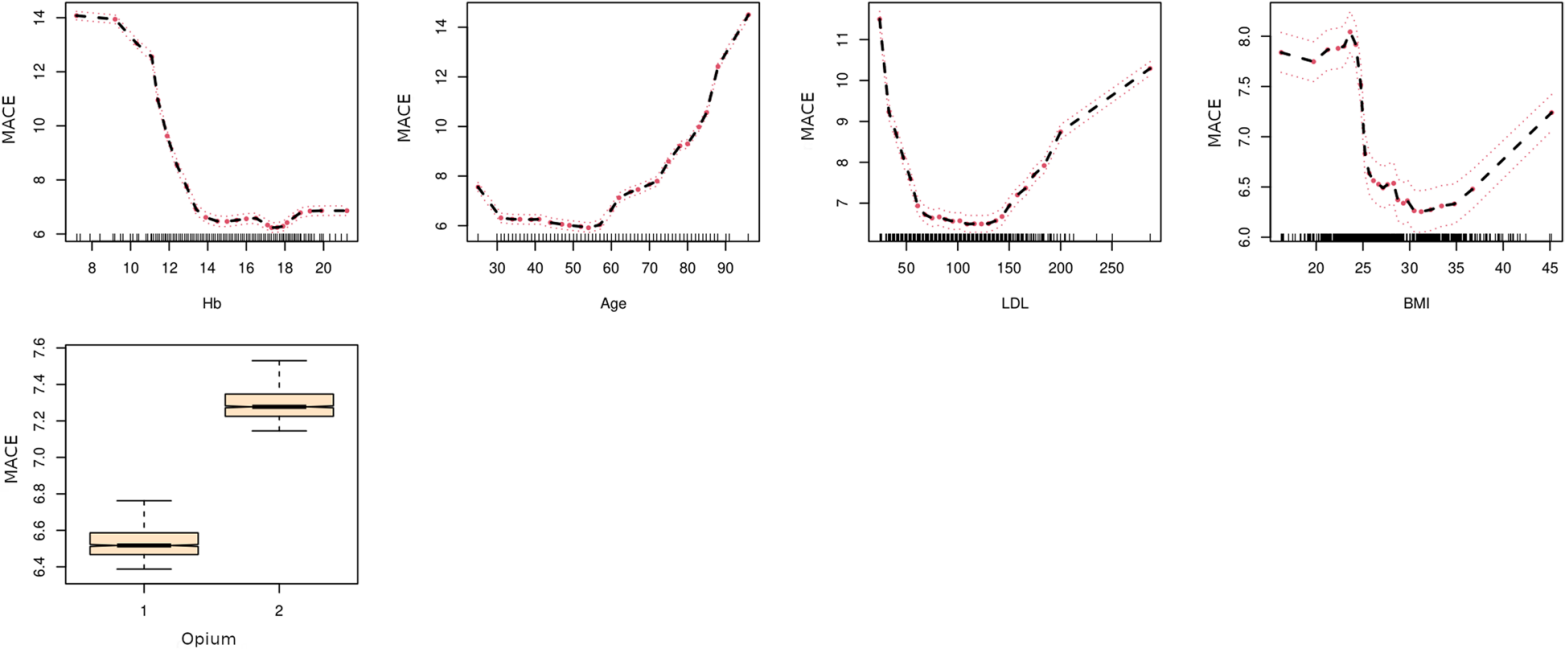
Partial dependence plot of MACE and top four variables plus opium. According to the plot, opium users had higher one-year MACE rates than non-opium users

On variable importance performed on out of the box (OOB) samples, opium use had positive VIMP score and ranked 13th among other variables (Fig. 4). Opium use also ranked 13th by minimal depth method (Fig. 5); and therefore, the results in variable importance and feature selection were concordant (Fig. 6). Opium use was ranked 12th in the variable hunting method (Fig. 7). Figure 8 Partial dependence plots illustrates marginal effects of opium use and four top variables example on one-year MACE. The plot shows that opium users had increased MACE compared with non-opium users.

Nelson-Aaren estimator and KM curves demonstrated nearly similar overall survival curves. Continuous ranked probability score (CRPS) and Brier score plots over time in OOB subjects, demonstrated acceptable prediction accuracy of the SRF model over time (Supplementary Fig. 6).

#### XGboost results

**Fig. 9:**
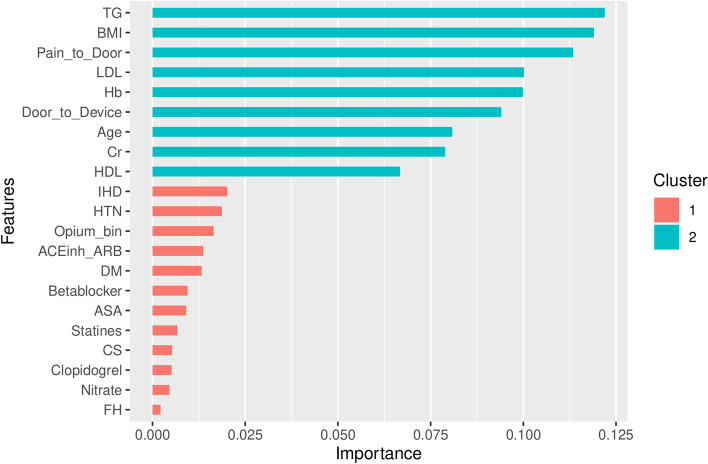
Variable importance analysis by XGboost algorithm

Opium ranked 12th among other variables using its built-in variable importance method on the training set (Fig. 9).

#### Performance analysis

Benchmarking on train dataset with nested (repeated) cross validation resampling demonstrated that random forest method outperformed cox proportional hazards (conventional analysis) and XGboost. XGboost had the lowest performance, although the performance of the methods did not differ much (Harrell’s C-index: random forest: 63.0%, Cox proportional hazards: 61.2%, XGboost: 59.2%) (Supplementary Table 2, Supplementary Fig. 7).

On the unseen test set, random forest model achieved a Harrell’s C-index of about 69.4%, about 7% more than the observed value in benchmarking. XGboost Harrell’s C-index value was similar to its value on the benchmarking with about 60%. Cox proportional hazard analysis was also performed on train and test dataset with all the independent variables. Its Harrell’s C-index was 66%.

**Fig. 10:**
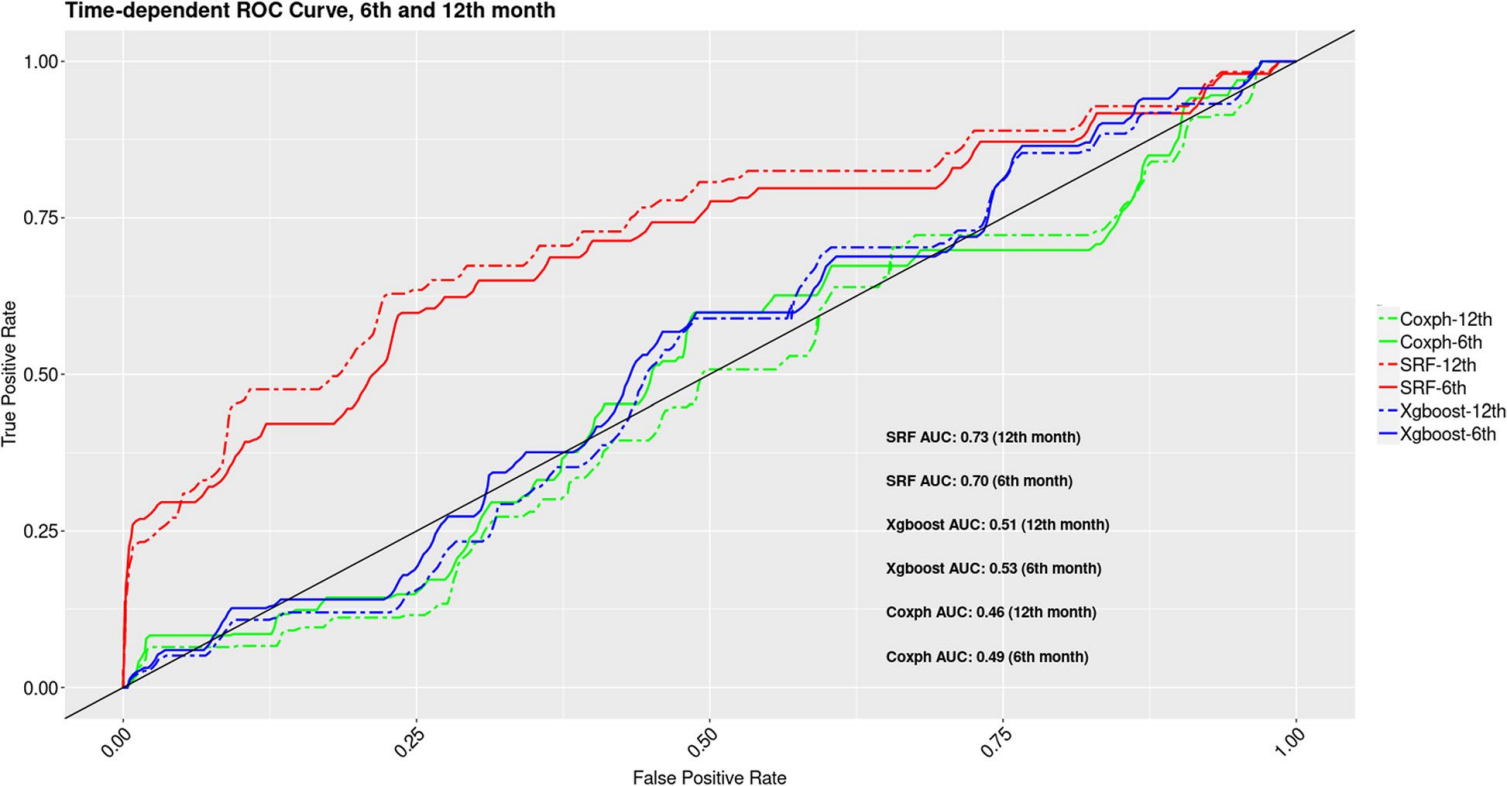
Time-dependant ROC (receiver operating characteristic) curve at 6 and 12 months for each model

Values of Harrell’s C-index between survival random forest and the two other models were significantly different, while XGboost and Coxph models were not significantly different regarding their Harrell’s C-index (Table 5). We then performed a time-dependent ROC analysis and assessed ROC AUC at six and 12 months after each model. As it is evident, at these time points, survival random forest remarkably outperformed other two models (Fig. 10).

**Table 5:** Comparing Harrell’s C-index between the models

Models	Z-score	***p***-value
XGboost vs SRF	2.37	0.02*
XGboost vs Coxph	−.0.99	0.32
SRF vs Coxph	−3.04	0.002*

* Two-sided *p*-value of <0.05 is considered as significant

## Discussion

Overall, this study suggests that opium use offers neither benefits nor strongly affirmed detrimental effect on the rate of MACE during first year following primary PCI, although our results arose possibilities of detrimental effects of opium after primary PCI. Opium use ranked 12 or 13 in the machine learning analyses and was not among the most influential risk factors (ie. top ten variables). Despite the fact that opium use demonstrated a trend towards higher one-year MACE in the conventional statistical analysis, the difference was not significant.

In our study we tried to make opium user and control groups as homogeneous as possible in terms of baseline features, especially those features having known major contribution to cardiovascular events. One main drawback of using variable ranking by machine learning models is that such methods do not consider confounding factors and do not adjust and control possible contribution of other variables to the outcome. Hence, we cannot ascertain that opium has independent adverse effect on first year MACE following primary PCI.

This finding is important, as many opium users believe in opium’s protective effects against several diseases and rely on this factor as a motivation for continued consumption. For example, a study in Iran found that 78.3% of the opium users believe that opium has positive effects in glycemic and hypertension control [4], while no such benefits were observed in the studies [5].

No previous study examined the association between opium use and primary PCI outcomes. A retrospective cohort in our center did not find associations between opium use and one-year MACE in males undergoing “elective” PCI, although the authors did not adjust the groups’ age as a potential confounder [20]. Mousavi et al. also found no increased in-hospital and six-month adverse outcomes after thrombolytic therapy for STEMI in patients addicted to opium compared to controls [21]. However, several studies on stable coronary artery disease (CAD), including a meta-analysis [6], found that opium use positively correlated with the risk of developing atherosclerotic plaque and CAD, the severity, and the risk of mortality from CAD [6–8, 10–12]. Dose-response associations were observed between opium use and the extent of atherosclerotic plaques according to Gensini’s score [10], CAD severity by clinical vessel score [11], and cardiovascular and all-cause mortality [9]. Furthermore, Sadeghian et al. reported opium use as the most important risk factor for premature CAD (< 45 years) among Iranian males [12]. Regarding acute coronary events (ACS), Roayaei et al. concluded that disagreements existed if opium had adverse effects on patients’ outcomes; however, at least no studies reported protective properties for opium [1].

Unlike primary PCI, outcomes were studied in opium users undergoing CABG. Masoudkabir et al. found higher 5-year mortality and MACE in patients who continued to use opium after CABG, but no such findings were observed in patients who quit opium use after their surgery [14]. Concurrently, Safaei et al. reported higher readmission rates in opium users following CABG compared to non-opium users [13].

Roayaei [1], Masoudkabir [5], and Nakhaee et al. [22] reviewed several mechanisms that opium can exert its detrimental effects on the cardiovascular system: [1] Increased inflammatory cytokines and decreased anti-inflammatory mediators, [2] elevated oxidative stress, [3] increased levels of pro-coagulant molecules, [4] higher rates of insulin resistance and metabolic syndrome [5, 23] altered hormone levels, notably decreased testosterone, estrogen, and adiponectin levels and hyperprolactinemia, [6] increased homocysteine levels, [7] physical inactivity and sedentary life style, [8] altered pain sensation and delayed clinical presentations leading to adverse outcomes, [9] other impurities and substances, most notably lead, and [10] interference with some antiplatelet medications, including aspirin, clopidogrel, prasugrel, and ticagrelor. As mentioned, opium interferes with antiplatelets and this may increase the risk of coronary and stent thrombosis. Therefore, appropriate studies on antiplatelet dosage modification may address this issue in patients who use opium. Neovascularization and collateral formation might also play role in the effects of opium on patients with cardiovascular disorders, as these mechanisms decrease the damage from acute and subacute ischemic events to the heart [24]. Opioids probably have pro-angiogenic properties that may hypothetically increase collateral coronary arteries [25], but may not be important regarding several mechanisms disfavoring opium use.

As mentioned earlier, opium using had no significant effect on MACE and its components among STEMI patients despite it has shown increasing all-cause mortality among post CABG patients. We hypothesize that this finding may be due to the already intensely high inflammatory state during STEMI active phase compared to less intense chronic pro-inflammatory effect of opium which would contribute to small portion of inflammatory milieu during STEMI. Also, relatively lower overall risk factors and lower coronary artery disease burden in STEMI patients, would represent lower chronic inflammatory state compared to those who need CABG. This makes CABG patients more likely to experience mortality due to various cause [26].

This study comes with some limitations. One is the retrospective nature of this cohort. Furthermore, we did not divide the patients to former and current opium users due to the lack of a universal definition and the design of our database. Another drawback is that machine learning methods cannot adjust for confounding variables that could alter the observed outcomes. We recommend future researchers to conduct studies that compare former and current patients who use opium. On the other hand, in our opinion, the novelty of this study and its robust statistical methods may compensate for its shortcoming.

## Conclusion

Opium had neither protective effects nor strongly affirmed detrimental effect on one-year MACE after primary PCI on patients presenting with STEMI. It was not ranked among top ten important variables in machine learning algorithms and had not significant effect in conventional statistical analysis on one-year MACE outcome despite adjusting for other variables. Accordingly, it could emphasize that treatment strategies for patients presenting with ST elevation MI should not be different for those who are opium users vs. non users, a point that can be studied in the future and mentioned in future STEMI guidelines. On the other hand, patients who believe opium has certain health benefits and is useful after primary PCI, should be counseled about the lack of evidence for such claims and the possible adverse effects of opium.

## Supplementary Information

**Additional file 1:** **Supplementary Table 1.** Baseline characteristics of the study groups before and after matching.

**Additional file 2:** **Supplementary Table 2.** Comparison of the results of two different machine learning models (Random Forest and XGboost) and cox proportional hazards (coxph) based on Uno and Harrell’s C-index. Resampling method was cross-validation for all the learners.

**Additional file 3: Supplementary Table 3.** Source codes for the application and analyses.

**Additional file 4: Supplementary Figure 1.** Absolute standardized mean difference between opium users and controls before and after propensity score matching (PSM).

**Additional file 5: Supplementary Figure 2. **Correlation matrix of the independent numerical variables.

**Additional file 6: Supplementary Figure 3.** Examples of plotted decision trees. Above decision tree is plotted using random forest and the bottom using extended gradient boosting (XGboost).

**Additional file 7: Supplementary Figure 4.** Out-of-box (OOB) error rates for MACE per number of tree.

**Additional file 8: Supplementary Figure 5.** Kaplan–Meier (KM) curves for the components of one-year MACE of the patients who underwent primary PCI after ST-segment elevation MI separated by opium users and controls.

**Additional file 9: Supplementary Figure 6.** Out-of-bag (OOB) survival plot for individuals, Brier score, and continuous ranked probability score (CRPS) plots. The top left plot illustrates Kaplan Meier (KM) plots for OOB sample of each individual, and also included aggregate KM results in and Nelson-Aalen estimator in green. Both methods show same survival curve. Top right plot illustrates OOB Brier score to assess accuracy of the predictions over time in quarters of patients. Less Brier score indicates better prediction. Bottom left plot shows CRPS over time, another measure of prediction accuracy. Bottom right plot shows individual subjects’ MACE outcome vs. time.

**Additional file 10: Supplementary Figure 7.** Box plot of performance comparison of three algorithms (Forest plot, XGboost, cox proportional hazards) based on Harrell’s C-index.

## Data Availability

The datasets generated and analyzed during the current study are not publicly available due to our institutional policies, but are available from the corresponding author on reasonable request.

## Abbreviations

CI: Confidence interval
CAD: Coronary artery disease
CABG: Coronary artery bypass graft
MACE: Major adverse cardiovascular events
PCI: Percutaneous coronary intervention
STEMI: ST-segment elevation myocardial infarction
PSM: Propensity score matching
DM: Diabetes mellitus
LDL: Low density lipoprotein
SMD: Standardized mean difference
IQR: Interquartile range
HR: Hazard ratio
KM: Kaplan-Meier
TVR: Target vessel revascularization
TLR: Target lesion revascularization
AUC: Area under the curve
VIMP: Variable importance
VH: Variable hunting
OOB: Out of the box
CRPS: Continuous ranked probability score
PDP: Partial dependance plot.
