# Supplementary material for: Effects of opium use on one-year major adverse cardiovascular events (MACE) in the patients with ST-segment elevation MI undergoing primary PCI: a propensity score matched - machine learning based study
Source: BMC Complement Med Ther. 2023 Jan 19;23:16. doi: 10.1186/s12906-023-03833-z (PMC9854103; doi:10.1186/s12906-023-03833-z)
Supplement: Supplementary file 1 — Additional file 1: Supplementary Table 1. Baseline characteristics of the study groups before and after matching. [file 12906_2023_3833_MOESM1_ESM.docx]

**Supplementary Table 1.** Baseline characteristics of the study groups before and after matching

|  |  | **Before Matching** | | |  | **After Matching** | | |
| --- | --- | --- | --- | --- | --- | --- | --- | --- |
| Variable | Category | Opium group | Control group | p-value |  | Opium group | Control group | p-value |
| Pain to Door time |  | 289 (IQR: 130.25 - 597.5 ) | 271.5 (IQR: 120 - 600) | 0.245 |  | 289 (IQR: 130.25 - 597.5 ) | 271.5 (IQR: 113.75 - 585) | 0.104 |
|  |  |  |  |  |  |  |  |  |
| Calcium channel blocker use |  |  |  | 0.372 |  |  |  | 0.268 |
|  | No | n= 495 (95.6%) | n= 2691 (94.5%) |  |  | n= 495 (95.6%) | n= 1003 (96.8%) |  |
|  | Yes | n= 23 (4.4%) | n= 157 (5.5%) |  |  | n= 23 (4.4%) | n= 33 (3.2%) |  |
|  |  |  |  |  |  |  |  |  |
| Nitrate use |  |  |  | 0.083 |  |  |  | 0.082 |
|  | No | n= 332 (64.1%) | n= 1939 (68.1%) |  |  | n= 332 (64.1%) | n= 711 (68.6%) |  |
|  | Yes | n= 186 (35.9%) | n= 909 (31.9%) |  |  | n= 186 (35.9%) | n= 325 (31.4%) |  |
|  |  |  |  |  |  |  |  |  |
| HDL |  | 37 (IQR: 31 - 43 ) | 37 (IQR: 32 - 43 ) | **0.029*** |  | 37 (IQR: 31 - 43 ) | 36 (IQR: 31 - 41 ) | 0.368 |
|  |  |  |  |  |  |  |  |  |
| FBS |  | 106 (IQR: 93 - 136 ) | 115 (IQR: 98 - 157 ) | **<0.001*** |  | 106 (IQR: 93 - 136 ) | 111 (IQR: 96 - 137.5 ) | 0.076 |
|  |  |  |  |  |  |  |  |  |
| Total cholesterol |  | 151 (IQR: 128 - 176 ) | 158 (IQR: 132 - 183 ) | **<0.001*** |  | 151 (IQR: 128 - 176 ) | 155 (IQR: 128 - 177.25 ) | 0.277 |
|  |  |  |  |  |  |  |  |  |
| LDL |  | 94 (IQR: 74 - 120.75 ) | 101 (IQR: 79 - 122 ) | **0.003*** |  | 94 (IQR: 74 - 120.75 ) | 100 (IQR: 76 - 119 ) | 0.38 |
|  |  |  |  |  |  |  |  |  |
| Triglyceride |  | 116 (IQR: 86 - 160.75 ) | 125 (IQR: 91 - 173 ) | **0.003*** |  | 116 (IQR: 86 - 160.75 ) | 125 (IQR: 91 - 172 ) | **0.016*** |
|  |  |  |  |  |  |  |  |  |
| BMI |  | 27 (IQR: 24.19 - 29.41 ) | 27.56 (IQR: 25.26 - 30.44 ) | **<0.001*** |  | 27 (IQR: 24.19 - 29.41 ) | 27.11 (IQR: 24.91 - 29.73 ) | **0.019*** |
|  |  |  |  |  |  |  |  |  |
| Creatinine |  | 1 (IQR: 0.8 - 1.02 ) | 0.9 (IQR: 0.8 - 1.1 ) | **0.001*** |  | 1 (IQR: 0.8 - 1.02 ) | 0.9 (IQR: 0.8 - 1.1 ) | **<0.001*** |
|  |  |  |  |  |  |  |  |  |
| Hemoglobin |  | 15 (IQR: 14.1 - 16.3 ) | 15.1 (IQR: 13.8 - 16.3 ) | 0.073 |  | 15 (IQR: 14.1 - 16.3 ) | 15.7 (IQR: 14.6 - 16.7 ) | **<0.001*** |
|  |  |  |  |  |  |  |  |  |
| Statin use |  |  |  | 0.568 |  |  |  | 0.318 |
|  | No | n= 289 (55.8%) | n= 1547 (54.3%) |  |  | n= 289 (55.8%) | n= 607 (58.6%) |  |
|  | Yes | n= 229 (44.2%) | n= 1301 (45.7%) |  |  | n= 229 (44.2%) | n= 429 (41.4%) |  |
|  |  |  |  |  |  |  |  |  |
| COPD |  |  |  | 0.742 |  |  |  | 0.414 |
|  | No | n= 508 (98.1%) | n= 2802 (98.4%) |  |  | n= 508 (98.1%) | n= 1023 (98.7%) |  |
|  | Yes | n= 10 (1.9%) | n= 46 (1.6%) |  |  | n= 10 (1.9%) | n= 13 (1.3%) |  |
|  |  |  |  |  |  |  |  |  |
| Aspirin use |  |  |  | 0.62 |  |  |  | 0.399 |
|  | No | n= 241 (46.5%) | n= 1362 (47.8%) |  |  | n= 241 (46.5%) | n= 507 (48.9%) |  |
|  | Yes | n= 277 (53.5%) | n= 1486 (52.2%) |  |  | n= 277 (53.5%) | n= 529 (51.1%) |  |
|  |  |  |  |  |  |  |  |  |
| Beta blocker use |  |  |  | 0.906 |  |  |  | 0.132 |
|  | No | n= 313 (60.4%) | n= 1732 (60.8%) |  |  | n= 313 (60.4%) | n= 668 (64.5%) |  |
|  | Yes | n= 205 (39.6%) | n= 1116 (39.2%) |  |  | n= 205 (39.6%) | n= 368 (35.5%) |  |
|  |  |  |  |  |  |  |  |  |
| ACEI/ARB use |  |  |  | 0.221 |  |  |  | 0.133 |
|  | No | n= 278 (53.7%) | n= 1442 (50.6%) |  |  | n= 278 (53.7%) | n= 599 (57.8%) |  |
|  | Yes | n= 240 (46.3%) | n= 1406 (49.4%) |  |  | n= 240 (46.3%) | n= 437 (42.2%) |  |
|  |  |  |  |  |  |  |  |  |
| Hypertension |  |  |  | **<0.001*** |  |  |  | 0.895 |
|  | No | n= 341 (65.8%) | n= 1505 (52.8%) |  |  | n= 341 (65.8%) | n= 677 (65.3%) |  |
|  | Yes | n= 177 (34.2%) | n= 1343 (47.2%) |  |  | n= 177 (34.2%) | n= 359 (34.7%) |  |
|  |  |  |  |  |  |  |  |  |
| DM |  |  |  | **<0.001*** |  |  |  | 1 |
|  | No | n= 354 (68.3%) | n= 1623 (57%) |  |  | n= 354 (68.3%) | n= 708 (68.3%) |  |
|  | Yes | n= 164 (31.7%) | n= 1225 (43%) |  |  | n= 164 (31.7%) | n= 328 (31.7%) |  |
|  |  |  |  |  |  |  |  |  |
| Hyperlipidemia |  |  |  | **<0.001*** |  |  |  | 0.732 |
|  | No | n= 292 (56.4%) | n= 1306 (45.9%) |  |  | n= 292 (56.4%) | n= 573 (55.3%) |  |
|  | Yes | n= 226 (43.6%) | n= 1542 (54.1%) |  |  | n= 226 (43.6%) | n= 463 (44.7%) |  |
|  |  |  |  |  |  |  |  |  |
| Family history of MI |  |  |  | 0.944 |  |  |  | 0.716 |
|  | No | n= 430 (83%) | n= 2371 (83.3%) |  |  | n= 430 (83%) | n= 869 (83.9%) |  |
|  | Yes | n= 88 (17%) | n= 477 (16.7%) |  |  | n= 88 (17%) | n= 167 (16.1%) |  |
|  |  |  |  |  |  |  |  |  |
| IHD |  |  |  | 0.201 |  |  |  | 0.066 |
|  | No | n= 352 (68%) | n= 2018 (70.9%) |  |  | n= 352 (68%) | n= 752 (72.6%) |  |
|  | Yes | n= 166 (32%) | n= 830 (29.1%) |  |  | n= 166 (32%) | n= 284 (27.4%) |  |
|  |  |  |  |  |  |  |  |  |
| Final TIMI2 score |  |  |  | 0.841 |  |  |  | 1 |
|  | 0 | n= 39 (7.5%) | n= 225 (7.9%) |  |  | n= 39 (7.5%) | n= 79 (7.6%) |  |
|  | 1 | n= 479 (92.5%) | n= 2623 (92.1%) |  |  | n= 479 (92.5%) | n= 957 (92.4%) |  |
|  |  |  |  |  |  |  |  |  |
| Gender |  |  |  | **<0.001*** |  |  |  | 0.315 |
|  | Female | n= 19 (3.7%) | n= 731 (25.7%) |  |  | n= 19 (3.7%) | n= 27 (2.6%) |  |
|  | Male | n= 499 (96.3%) | n= 2117 (74.3%) |  |  | n= 499 (96.3%) | n= 1009 (97.4%) |  |
|  |  |  |  |  |  |  |  |  |
| Age |  | 58 (IQR: 52 - 65 ) | 61 (IQR: 53 - 69 ) | **<0.001*** |  | 58 (IQR: 52 - 65 ) | 58 (IQR: 51 - 65.25 ) | 0.963 |
|  |  |  |  |  |  |  |  |  |
| Chronic smoking |  |  |  | **<0.001*** |  |  |  | 0.659 |
|  | No | n= 91 (17.6%) | n= 1758 (61.7%) |  |  | n= 91 (17.6%) | n= 193 (18.6%) |  |
|  | Yes | n= 427 (82.4%) | n= 1090 (38.3%) |  |  | n= 427 (82.4%) | n= 843 (81.4%) |  |
|  |  |  |  |  |  |  |  |  |
| Block |  |  |  | 0.195 |  |  |  | 0.423 |
|  | FdAVB | n= 1 (0.2%) | n= 2 (0.1%) |  |  | n= 1 (0.2%) | n= 1 (0.1%) |  |
|  | LBBB | n= 2 (0.4%) | n= 38 (1.3%) |  |  | n= 2 (0.4%) | n= 9 (0.9%) |  |
|  | No | n= 501 (96.7%) | n= 2713 (95.3%) |  |  | n= 501 (96.7%) | n= 986 (95.2%) |  |
|  | RBBB | n= 14 (2.7%) | n= 95 (3.3%) |  |  | n= 14 (2.7%) | n= 40 (3.9%) |  |
|  |  |  |  |  |  |  |  |  |
| Initial TIMI2 score |  |  |  | 0.171 |  |  |  | 0.087 |
|  | 0 | n= 315 (60.8%) | n= 1735 (60.9%) |  |  | n= 315 (60.8%) | n= 637 (61.5%) |  |
|  | 1 | n= 32 (6.2%) | n= 253 (8.9%) |  |  | n= 32 (6.2%) | n= 99 (9.6%) |  |
|  | 2 | n= 115 (22.2%) | n= 569 (20%) |  |  | n= 115 (22.2%) | n= 202 (19.5%) |  |
|  | 3 | n= 56 (10.8%) | n= 291 (10.2%) |  |  | n= 56 (10.8%) | n= 98 (9.5%) |  |
|  |  |  |  |  |  |  |  |  |
| PCI result |  |  |  | 0.868 |  |  |  | 1 |
|  | Successful | n= 489 (94.4%) | n= 2680 (94.1%) |  |  | n= 489 (94.4%) | n= 978 (94.4%) |  |
|  | Unacceptable | n= 29 (5.6%) | n= 168 (5.9%) |  |  | n= 29 (5.6%) | n= 58 (5.6%) |  |
|  |  |  |  |  |  |  |  |  |
| Culprit vessel |  |  |  | 0.739 |  |  |  | 0.455 |
|  | LM LADp | n= 150 (29%) | n= 778 (27.3%) |  |  | n= 150 (29%) | n= 274 (26.4%) |  |
|  | LADnp | n= 124 (23.9%) | n= 691 (24.3%) |  |  | n= 124 (23.9%) | n= 241 (23.3%) |  |
|  | Non-LAD | n= 244 (47.1%) | n= 1379 (48.4%) |  |  | n= 244 (47.1%) | n= 521 (50.3%) |  |
|  |  |  |  |  |  |  |  |  |
| IABP |  |  |  | 0.658 |  |  |  | 0.54 |
|  | No | n= 516 (99.6%) | n= 2843 (99.8%) |  |  | n= 516 (99.6%) | n= 1035 (99.9%) |  |
|  | Yes | n= 2 (0.4%) | n= 5 (0.2%) |  |  | n= 2 (0.4%) | n= 1 (0.1%) |  |
|  |  |  |  |  |  |  |  |  |
| Cardiogenic shock |  |  |  | 0.951 |  |  |  | 0.724 |
|  | No | n= 517 (99.8%) | n= 2846 (99.9%) |  |  | n= 517 (99.8%) | n= 1036 (100%) |  |
|  | Yes | n= 1 (0.2%) | n= 2 (0.1%) |  |  | n= 1 (0.2%) | n= 0 (0%) |  |
|  |  |  |  |  |  |  |  |  |
| Door to Device time |  | 52 (IQR: 39 - 79.5 ) | 53 (IQR: 36 - 90 ) | 0.773 |  | 52 (IQR: 39 - 79.5 ) | 51 (IQR: 35 - 85 ) | 0.573 |
|  |  |  |  |  |  |  |  |  |
| GPIIbIIIa inhibitors use |  |  |  | 1 |  |  |  | 0.495 |
|  | No | n= 394 (76.1%) | n= 2168 (76.1%) |  |  | n= 394 (76.1%) | n= 770 (74.3%) |  |
|  | Yes | n= 124 (23.9%) | n= 680 (23.9%) |  |  | n= 124 (23.9%) | n= 266 (25.7%) |  |

**Abbreviations:** IQR: Interquartile range, HDL: High-density lipoprotein, FBS: Fasting blood sugar, LDL: Low-density lipoprotein, BMI: Body mass index, COPD: Chronic obstructive pulmonary disease, ACEI: Angiotensin-converting enzyme inhibitor, ARB: Angiotensin receptor blocker, DM: Diabetes mellitus, MI: Myocardial infarction, IHD: Ischemic heart disease, FdAVB: First-degree atrioventricular block, LBBB: Left bundle branch block, RBBB: Right bundle branch block, TIMI: Thrombolysis in Myocardial Infarction, PCI: Percutaneous coronary intervention, LM: Left main coronary artery, LADp: Proximal left anterior descending coronary artery, LADnp: Non-proximal LAD, IABP: Intra-aortic balloon pump

* Wald p-value of less than 0.1 was the cut-off point for univariate analysis. Significant level for the multivariate analysis was p < 0.05.
