# Supplementary material for: Effects of opium use on one-year major adverse cardiovascular events (MACE) in the patients with ST-segment elevation MI undergoing primary PCI: a propensity score matched - machine learning based study
Source: BMC Complement Med Ther. 2023 Jan 19;23:16. doi: 10.1186/s12906-023-03833-z (PMC9854103; doi:10.1186/s12906-023-03833-z)
Supplement: Supplementary file 2 — Additional file 2: Supplementary Table 2. Comparison of the results of two different machine learning models (Random Forest and XGboost) and cox proportional hazards (coxph) based on Uno and Harrell’s C-index. Resampling method was cross-validation for all the learners. [file 12906_2023_3833_MOESM2_ESM.docx]

**Supplementary Table 2.** Comparison of the results of two different machine learning models (Random Forest and XGboost) and cox proportional hazards (coxph) based on Uno and Harrell’s C-index. Resampling method was cross-validation for all the learners.

| **Learner** | **Iterations** | **Uno’ C** | **Harrell’s C** |
| --- | --- | --- | --- |
| Survival Random Forest | 10 | 0.6521540 | 0.6303952 |
| Xgboost (survival) | 10 | 0.5951234 | 0.5923385 |
| coxph | 10 | 0.6077105 | 0.6116708 |
