# Supplementary material for: Effects of opium use on one-year major adverse cardiovascular events (MACE) in the patients with ST-segment elevation MI undergoing primary PCI: a propensity score matched - machine learning based study
Source: BMC Complement Med Ther. 2023 Jan 19;23:16. doi: 10.1186/s12906-023-03833-z (PMC9854103; doi:10.1186/s12906-023-03833-z)
Supplement: Supplementary file 3 — Additional file 3: Supplementary Table 3. Source codes for the application and analyses. [file 12906_2023_3833_MOESM3_ESM.docx]

**Supplementary Table 3.** Source codes for the application and analyses

| Web app links | https://behnam-hedayat.shinyapps.io/primace |
| --- | --- |
|  | https://primace.aikadeh.com |
| Source code for the application | https://github.com/hedayatbehnam/primace |
| Source code for the analyses | https://github.com/hedayatbehnam/primace_study |
