# Supplementary material for: Effects of opium use on one-year major adverse cardiovascular events (MACE) in the patients with ST-segment elevation MI undergoing primary PCI: a propensity score matched - machine learning based study
Source: BMC Complement Med Ther. 2023 Jan 19;23:16. doi: 10.1186/s12906-023-03833-z (PMC9854103; doi:10.1186/s12906-023-03833-z)
Supplement: Supplementary file 4 — Additional file 4: Supplementary Figure 1. Absolute standardized mean difference between opium users and controls before and after propensity score matching (PSM). [file 12906_2023_3833_MOESM4_ESM.docx]

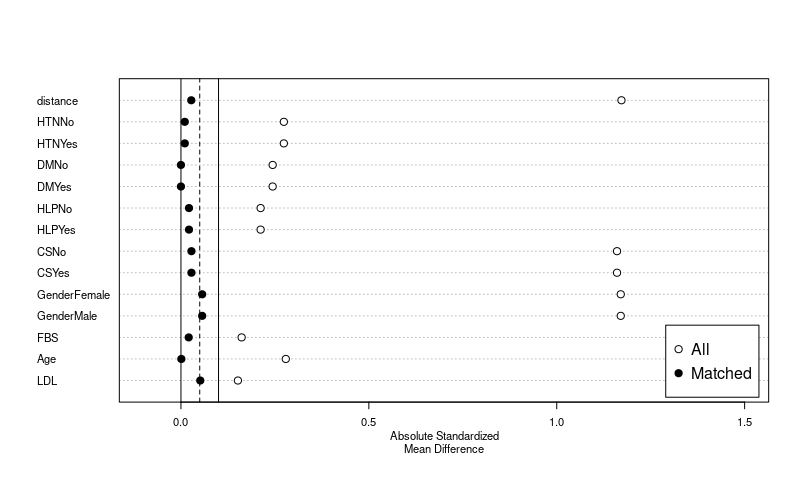


**Supplementary Figure 1.** Absolute standardized mean difference between opium users and controls before and after propensity score matching (PSM)
