# Supplementary figures and images for: Effects of opium use on one-year major adverse cardiovascular events (MACE) in the patients with ST-segment elevation MI undergoing primary PCI: a propensity score matched - machine learning based study
Source: BMC Complement Med Ther. 2023 Jan 19;23:16. doi: 10.1186/s12906-023-03833-z (PMC9854103; doi:10.1186/s12906-023-03833-z)

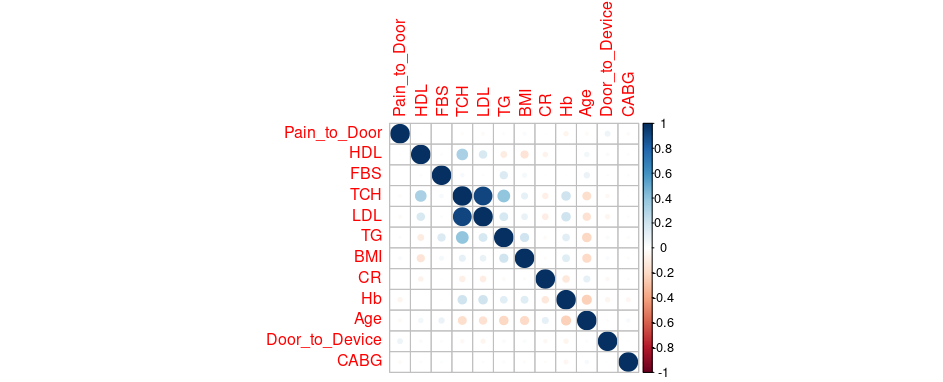


**Supplementary Figure 2.** Correlation matrix of the independent numerical variables

Supplement: Supplementary file 5 — Additional file 5: Supplementary Figure 2. Correlation matrix of the independent numerical variables. [file 12906_2023_3833_MOESM5_ESM.docx]

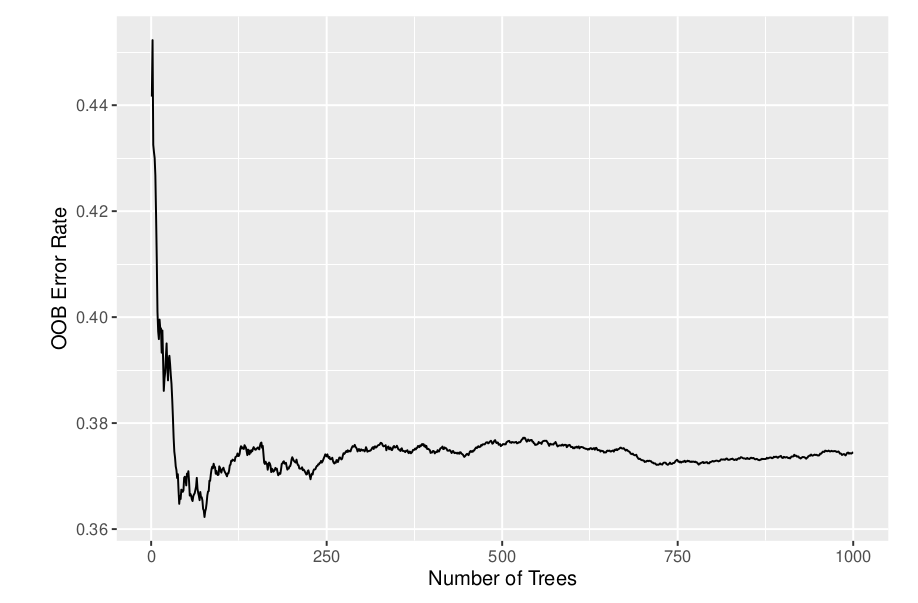


**Supplementary Figure 4.** Out-of-box (OOB) error rates for MACE per number of tree

Supplement: Supplementary file 7 — Additional file 7: Supplementary Figure 4. Out-of-box (OOB) error rates for MACE per number of tree. [file 12906_2023_3833_MOESM7_ESM.docx]
