# Supplementary material for: Effects of opium use on one-year major adverse cardiovascular events (MACE) in the patients with ST-segment elevation MI undergoing primary PCI: a propensity score matched - machine learning based study
Source: BMC Complement Med Ther. 2023 Jan 19;23:16. doi: 10.1186/s12906-023-03833-z (PMC9854103; doi:10.1186/s12906-023-03833-z)
Supplement: Supplementary file 6 — Additional file 6: Supplementary Figure 3. Examples of plotted decision trees. Above decision tree is plotted using random forest and the bottom using extended gradient boosting (XGboost). [file 12906_2023_3833_MOESM6_ESM.docx]

**
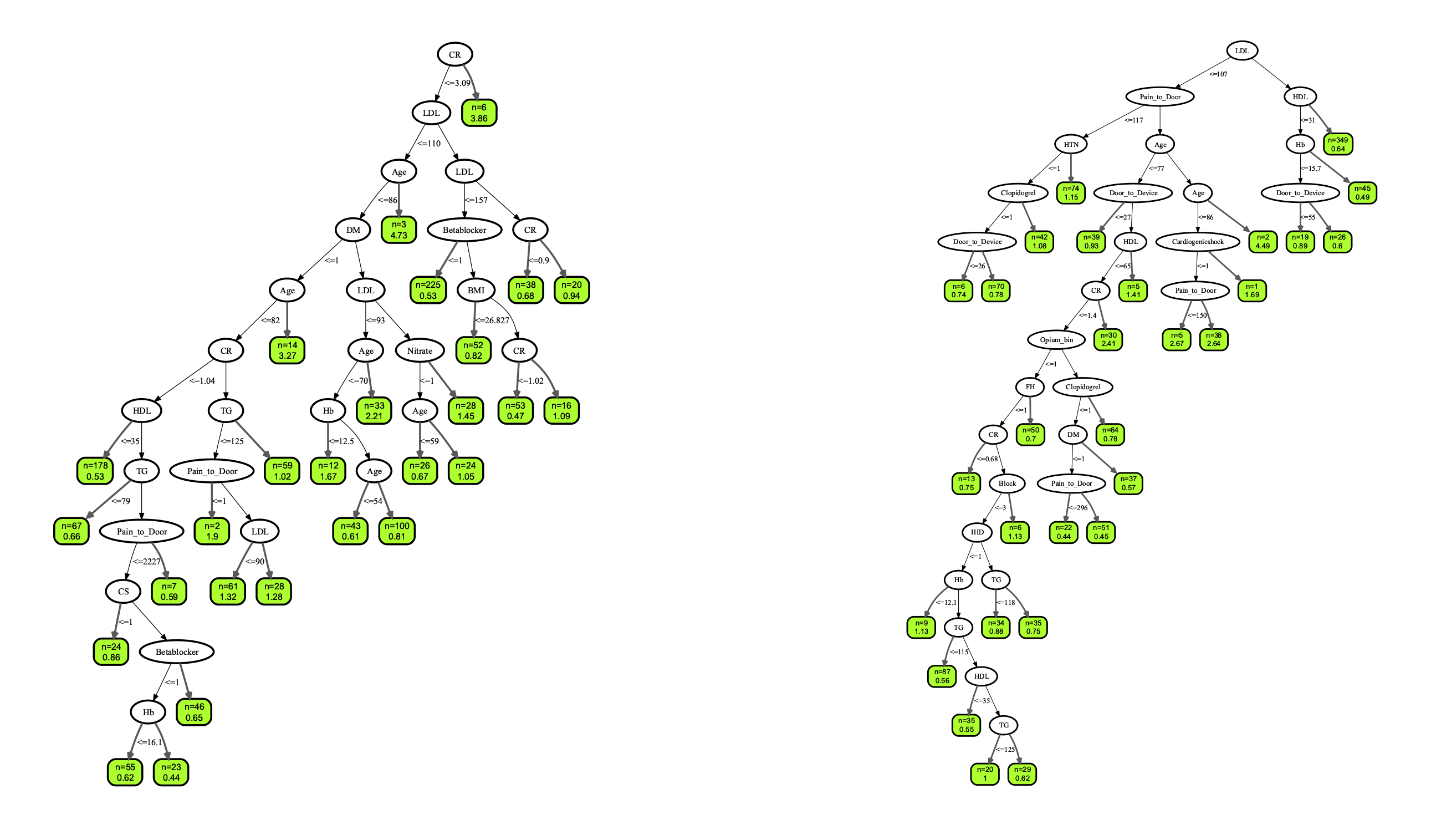
**

**
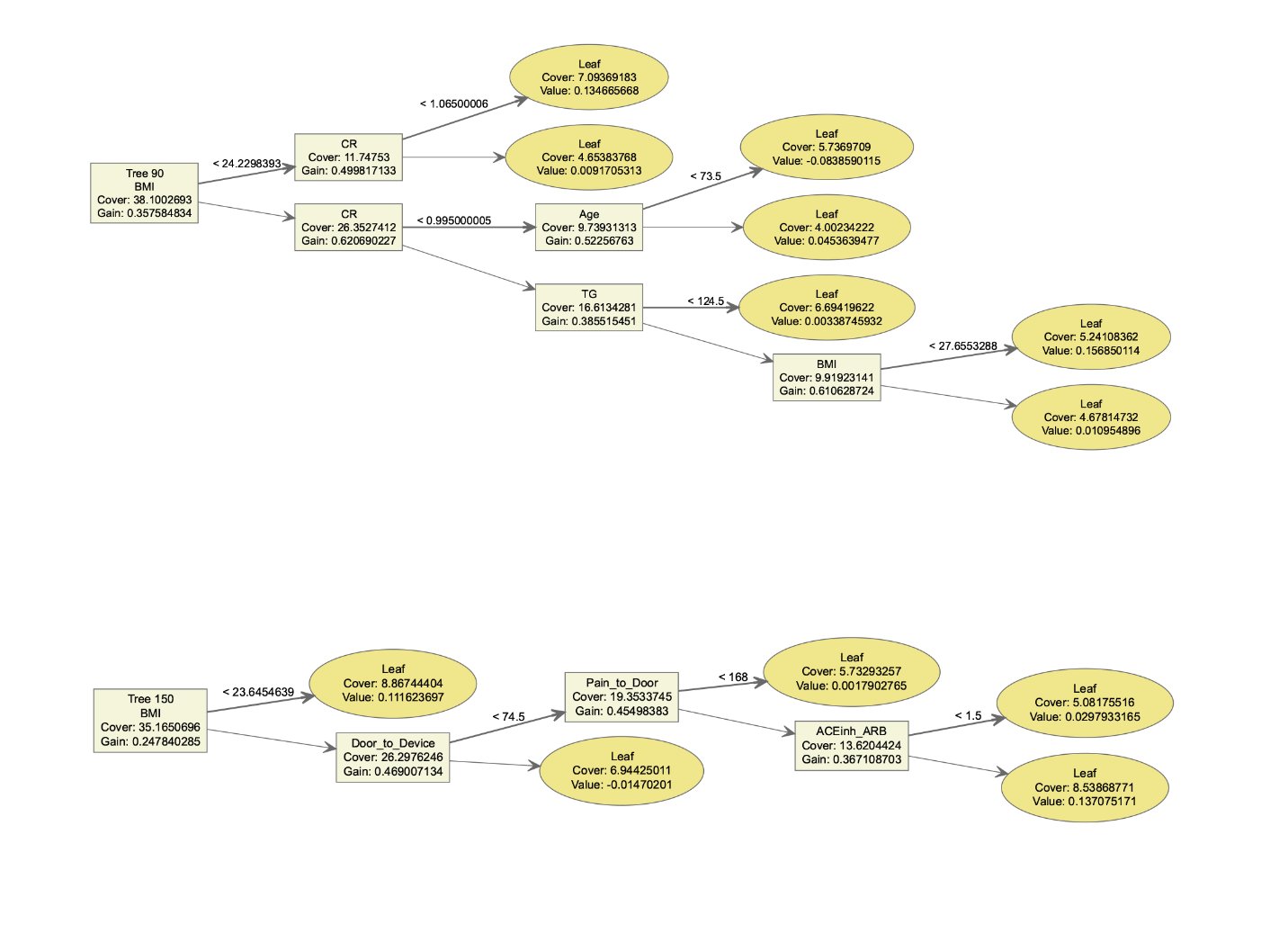
**

**Supplementary Figure 3.** Examples of plotted decision trees. Above decision tree is plotted using random forest and the bottom using extended gradient boosting (XGboost)
