# Supplementary material for: Effects of opium use on one-year major adverse cardiovascular events (MACE) in the patients with ST-segment elevation MI undergoing primary PCI: a propensity score matched - machine learning based study
Source: BMC Complement Med Ther. 2023 Jan 19;23:16. doi: 10.1186/s12906-023-03833-z (PMC9854103; doi:10.1186/s12906-023-03833-z)
Supplement: Supplementary file 8 — Additional file 8: Supplementary Figure 5. Kaplan–Meier (KM) curves for the components of one-year MACE of the patients who underwent primary PCI after ST-segment elevation MI separated by opium users and controls. [file 12906_2023_3833_MOESM8_ESM.docx]

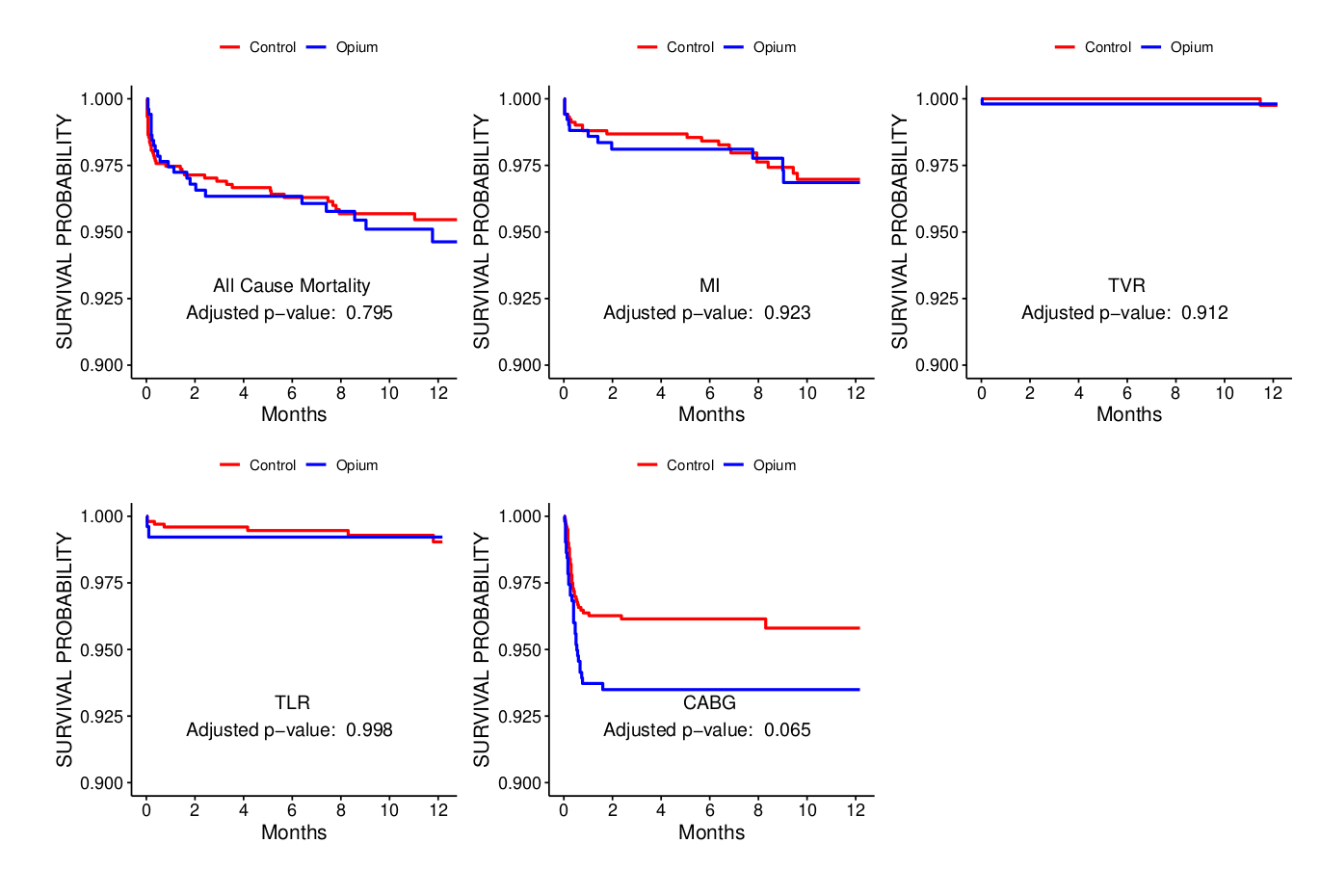


**Supplementary Figure 5.** Kaplan–Meier (KM) curves for the components of one-year MACE of the patients who underwent primary PCI after ST-segment elevation MI separated by opium users and controls.
