# Supplementary material for: Effects of opium use on one-year major adverse cardiovascular events (MACE) in the patients with ST-segment elevation MI undergoing primary PCI: a propensity score matched - machine learning based study
Source: BMC Complement Med Ther. 2023 Jan 19;23:16. doi: 10.1186/s12906-023-03833-z (PMC9854103; doi:10.1186/s12906-023-03833-z)
Supplement: Supplementary file 9 — Additional file 9: Supplementary Figure 6. Out-of-bag (OOB) survival plot for individuals, Brier score, and continuous ranked probability score (CRPS) plots. The top left plot illustrates Kaplan Meier (KM) plots for OOB sample of each individual, and also included aggregate KM results in and Nelson-Aalen estimator in green. Both methods show same survival curve. Top right plot illustrates OOB Brier score to assess accuracy of the predictions over time in quarters of patients. Less Brier score indicates better prediction. Bottom left plot shows CRPS over time, another measure of prediction accuracy. Bottom right plot shows individual subjects’ MACE outcome vs. time. [file 12906_2023_3833_MOESM9_ESM.docx]

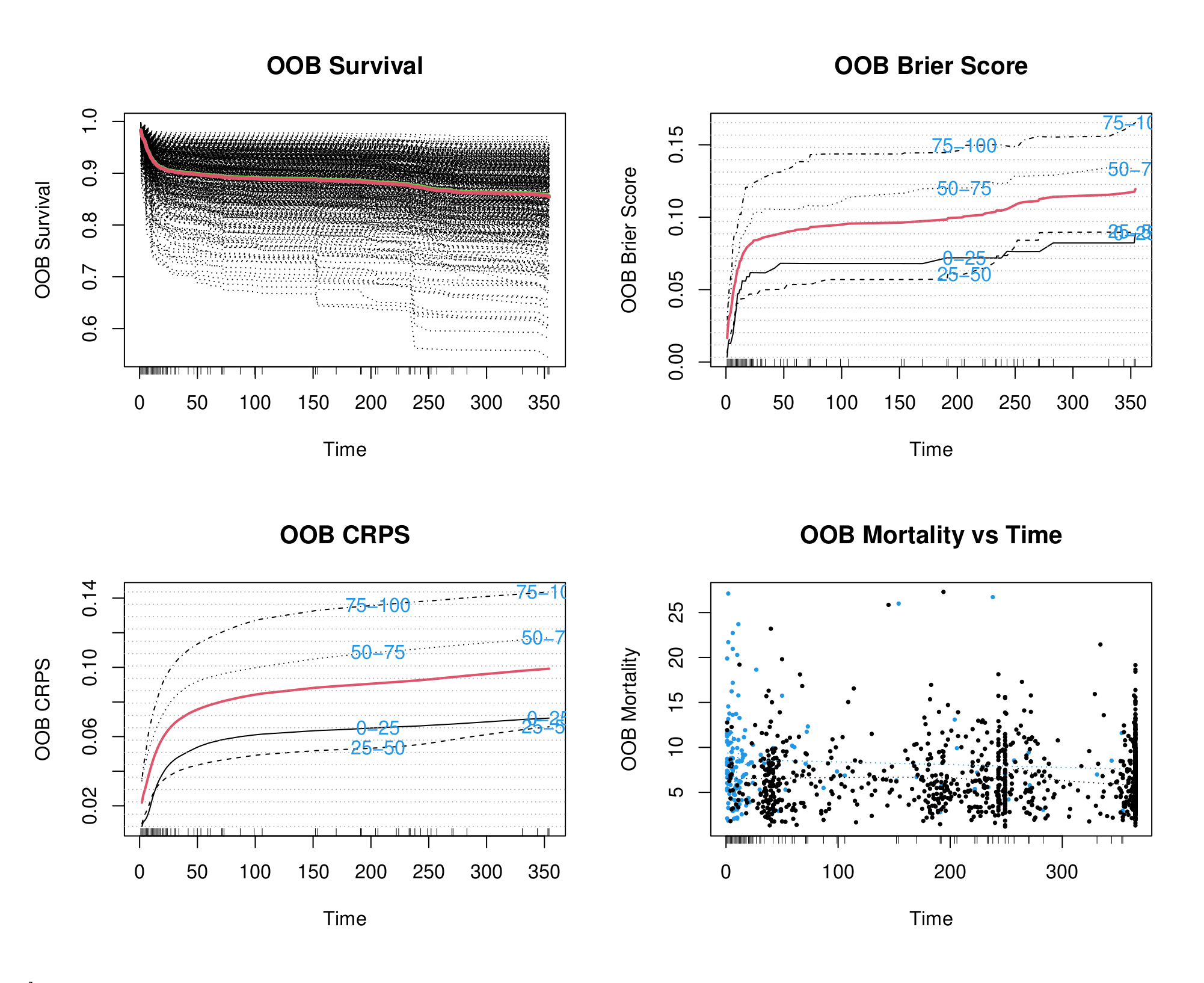


**Supplementary Figure 6.** Out-of-bag (OOB) survival plot for individuals, Brier score, and continuous ranked probability score (CRPS) plots. The top left plot illustrates Kaplan Meier (KM) plots for OOB sample of each individual, and also included aggregate KM results in and Nelson-Aalen estimator in green. Both methods show same survival curve. Top right plot illustrates OOB Brier score to assess accuracy of the predictions over time in quarters of patients. Less Brier score indicates better prediction. Bottom left plot shows CRPS over time, another measure of prediction accuracy. Bottom right plot shows individual subjects’ MACE outcome vs. time
